# Structural insights into polyamine spermidine uptake by the ABC transporter PotD-PotABC

**DOI:** 10.1126/sciadv.ado8107

**Published:** 2024-09-20

**Authors:** Zhu Qiao, Phong Hoa Do, Joshua Yi Yeo, Rya Ero, Zhuowen Li, Liying Zhan, Sandip Basak, Yong-Gui Gao

**Affiliations:** ^1^ School of Biological Sciences, Nanyang Technological University, Singapore 637551, Singapore.; ^2^ NTU Institute of Structural Biology, Nanyang Technological University, Singapore 636921, Singapore.; ^3^ Department of Critical Care Medicine, Renmin Hospital of Wuhan University, Wuhan 430060, China.

## Abstract

Polyamines, characterized by their polycationic nature, are ubiquitously present in all organisms and play numerous cellular functions. Among polyamines, spermidine stands out as the predominant type in both prokaryotic and eukaryotic cells. The PotD-PotABC protein complex in *Escherichia coli*, belonging to the adenosine triphosphate–binding cassette transporter family, is a spermidine-preferential uptake system. Here, we report structural details of the polyamine uptake system PotD-PotABC in various states. Our analyses reveal distinct “inward-facing” and “outward-facing” conformations of the PotD-PotABC transporter, as well as conformational changes in the “gating” residues (F222, Y223, D226, and K241 in PotB; Y219 and K223 in PotC) controlling spermidine uptake. Therefore, our structural analysis provides insights into how the PotD-PotABC importer recognizes the substrate-binding protein PotD and elucidates molecular insights into the spermidine uptake mechanism of bacteria.

## Teaser

Atomic structures of the bacterial spermidine transporter complex in various states elucidate the spermidine uptake mechanism.

## INTRODUCTION

Polyamines are polycation organic molecules that are universally present in the three domains of life (*1*). There are three primary types of polyamines: putrescine (Put), spermidine (Spd), and spermine (Spm). In prokaryotic cells, Put and Spd are the major types, while in eukaryotic cells, Spd and Spm are more dominant (*1*). Under physiological conditions, polyamines are fully protonated, enabling them to bind to biomolecules such as nucleic acids, proteins, and phospholipids (*1*, *2*). Polyamines are critical for cell survival and growth, in both prokaryotic and eukaryotic cells (*3*). In bacteria, Spd has been implicated in cell growth, motility, cell wall formation, biofilm development, biosynthesis of siderophores, and secretion system formation (*4*–*6*). In humans, polyamines are reported to be involved in diverse cellular functions, such as cell growth, proliferation, differentiation, gene re-gulation, signaling, and apoptosis (*7*, *8*). Polyamine concentration in tissues decreases with age, and a diet supplement with polyamines is suggested to be health-beneficial and extend the overall life span (*9*–*11*).

Given the vital functions of polyamines, it is imperative to regulate their cellular levels through processes such as biosynthesis, degradation, and transport (*1*). Put is synthesized from ornithine by ornithine decarboxylase (ODC); it can then be further modified to Spd and subsequently to Spm in sequential order, catalyzed by the spermidine synthase and spermine synthase, respectively (*3*, *12*). In addition, Spm can be reversibly converted to Spd by spermine oxidase or spermidine/spermine N1-acetyltransferase (SSAT) and acetylpolyamine oxidase (PAO), and Spd to Put by SSAT and PAO (*12*). Notably, in response to an increased cellular polyamine level in human cells, an enzyme antizyme, resulting from +1 ribosomal frameshifting of its mRNA, is synthesized to interact with ODC and trigger its degradation, thereby functioning as a negative regulator for polyamine biosynthesis (*13*). In contrast, such an antizyme regulation system has not been found in prokaryotic cells.

In addition to de novo biosynthesis, transport is another key pathway in maintaining cellular polyamine levels in both prokaryotes and eukaryotes. In the bacterial model organism *Escherichia coli* (*E. coli*), two distinct polyamine uptake systems have been identified to date, both falling under the category of adenosine triphosphate (ATP)–binding cassette (ABC) transporters. These include the Spd-preferential uptake complex PotD-PotABC and the Put-specific uptake complex PotF-PotGHI (*7*, *14*, *15*). For the former, PotABC forms the ABC transporter, while PotD is responsible for substrate binding. For the latter, PotGHI and PotF function as the ABC transporter and the substrate-binding protein, respectively. In general, ATP binding, hydrolysis, and phosphate release trigger conformational changes in the transporter, leading to the uptake of extracellular polyamines. Moreover, additional polyamine transporters, such as PotE, CadB, MdtI/J, and PuuP, have been identified in *E. coli* (*16*). No plasma membrane polyamine transporters have been identified in humans, and presumably, the polyamine uptake is mediated by endocytosis (*16*). ATP13A2 (PARK9), a late endolysosomal transporter, has been recently identified as a lysosomal polyamine exporter (*2*, *17*).

While it is crucial to maintain the homeostasis of cellular polyamines, the understanding of polyamine transport lags far behind compared to polyamine biosynthesis and regulation. In particular, the underlying molecular basis of polyamine uptake is still largely unknown. To date, only the structure of the lysosomal polyamine exporter ATP13A2, which is a single-protein P5-type adenosine triphosphatase (ATPase) in humans and different from bacterial polyamine transporters like PotD-PotABC and PotF-PotGHI complexes, has been characterized very recently by cryo–electron microscopy (cryo-EM) (*2*, *18*). Here, we report the cryo-EM structures of the *E. coli* Spd-preferential importer PotD-PotABC in various states that shed light on this elusive polyamine uptake system. Our structures of PotD-PotABC greatly enhance the understanding of polyamine transportation in bacteria and can aid in the discovery of drugs to fight pathogenic infections.

## RESULTS

### Structures of the apo- and Spd-bound PotABC complexes

To shed light on the polyamine transport processes in bacteria, we aimed to structurally characterize the various functional states of the *E. coli* PotABC protein complex, an ABC transporter with a preference for Spd over other types of polyamines (fig. S1A). The PotABC complex was successfully expressed and purified as described in Materials and Methods (fig. S1B). We introduced the E173Q mutation to PotA to stabilize the PotABC complex (fig. S1C), a similar strategy as described in previous studies (*19*–*21*) and used for our other structural work (*22*, *23*). In addition, we also reconstituted the wild-type PotABC complex into nanodiscs (fig. S1D). The purified wild-type PotABC complex exhibited strong ATPase activity, comparable to the previously characterized other type 1 ABC transporters (*20*, *21*), whereas the mutant PotABC E173Q exhibited only marginal ATPase activity (fig. S1E). The ATPase activity of PotABC was markedly reduced by the substrate Spd, which is consistent with the previous report (*24*). PotD can stimulate PotABC activity regardless of the presence or absence of Spd (fig. S1E). Next, to prepare the sample for cryo-EM structure determination, we incubated the PotABC E173Q complex with ATP, Mg^2+^, and Spd. Following data processing, we obtained high-resolution (~3 Å) electron density maps of the PotABC complex in the presence and absence (apo form) of Spd, enabling us to de novo build the atomic models (figs. S2 and S3, A and B, and table S1).

**Fig. 1.**
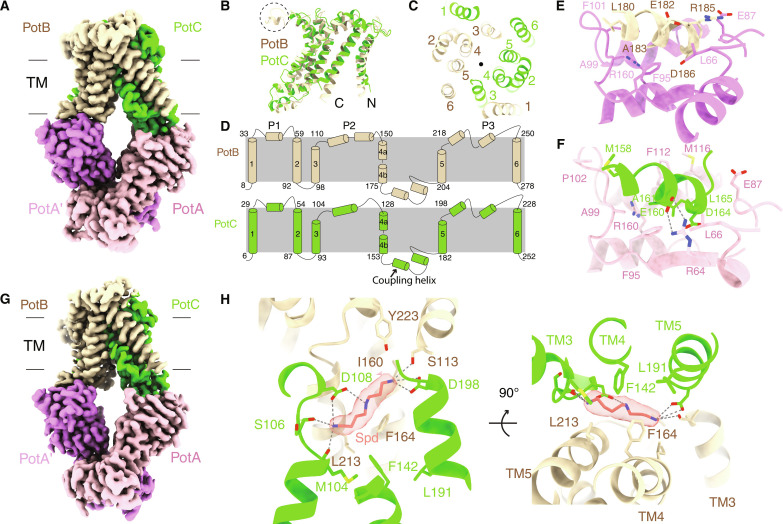
Structures of the apo form and Spd-bound PotABC complexes. (**A**) Cryo-EM map of the apo form PotABC complex. The two PotA molecules are colored pink (PotA) and purple (PotA′); the PotB and PotC molecules are colored wheat and lime, respectively. The protein molecules are colored consistently in all figures unless stated otherwise. The gray lines indicate the inner membrane location, and the transmembrane (TM) region is shown. (**B**) Cartoon representation of PotB and PotC alignment. The two termini are indicated by N and C. The extra helix in PotB is marked with a dashed circle. (**C**) PotB and PotC assemble with a twofold pseudosymmetry. The symmetry axis is indicated with a black dot, and the transmembrane helices are numbered as in (D). (**D**) Topological diagrams of PotB and PotC. The three periplasmic loops are indicated by P1, P2, and P3, and the coupling helix is indicated by an arrow. (**E**) Detailed interactions between the coupling helix of PotA and PotB. The interacting residues are shown as sticks, the same as the below. (**F**) Detailed interactions between the coupling helix of PotA and PotC. The hydrogen bonds are shown as dashed lines, the same as the ones below. (**G**) Cryo-EM map of the Spd-bound PotABC complex. (**H**) Two views showing the detailed interactions between Spd and PotB/PotC. Spd is shown in sticks and colored salmon, with the electron density shown as salmon surface. The residues (PotB F164 and L213; and PotC F142 and L191) blocking the exit of Spd from the membrane translocation pathway are also shown in sticks.

The overall architecture of the apo PotABC complex, consisting of two PotA subunits, one PotB, and one PotC, adopts an “inward-facing” conformation (Fig. 1A), as observed for the type 1 ABC transporter (*20*, *21*, *25*, *26*). In this complex, the two PotA subunits are assembled as a homodimer and located entirely in the cytosol, whereas PotB and PotC form a heterodimeric transmembrane complex, with both proteins contributing six transmembrane helices arranged with a twofold pseudosymmetry (Fig. 1, B and C). Despite a sequence identity of only 21.9% between PotB and PotC, their tertiary structures are overall similar, with a root mean square deviation (RMSD) of 3.97 Å for the 247 pairs of Cα atoms. The only notable variation lies in the periplasmic loop 2 (P2). Namely, PotB has one more helix (starting from Y129 to L137) in the P2 loop compared to PotC (Fig. 1, B and D). PotA, the cytosolic subunit of the complex, contains both the nucleotide-binding domain (NBD) and the regulatory domain. The NBD is capable of binding and hydrolyzing ATP, thereby providing the energy required for Spd uptake. In addition, it interacts with the “coupling helices” of PotB and PotC through a cleft located just beneath the inner membrane (Fig. 1, E and F).

We also successfully modeled the structure of the PotABC complex with the bound ligand Spd. The overall structures of the PotABC complex in both the apo- (Fig. 1A) and Spd-bound (Fig. 1G) states demonstrate a remarkable similarity, with an RMSD of 0.94 Å for all 1240 pairs of Cα atoms (fig. S3C). We observed a slight closure (by ~1.4 Å) of the transmembrane pathway in the cytosol side upon Spd binding (fig. S3D). In the Spd-bound complex, one Spd molecule is enclosed within the membrane cavity, formed mainly by transmembrane helices 3 to 5 (TM3 to TM5) of both PotB and PotC (Fig. 1H). Notably, the density map is clearly distinguishable, enabling us to unambiguously model the Spd rather than other bacterial polyamines (such as Put), given the different carbon chain length for polyamines and the present well-fitting of Spd into the network of interactions (Fig. 1H and fig. S1A). The residues S113 and Y223 in PotB, along with D198 in PotC, establish interactions with Spd amine at one end, while residues M104, S106, and D108 in PotC interact with the other end (Fig. 1H). D108 also interacts with the middle amine group. In addition, residues I160, F164, and L213 in PotB engage in hydrophobic interactions with the carbon atoms of Spd. Together, PotB (F164 and L213) and PotC (F142 and L191) block Spd exit into the cytosol (Fig. 1H). These intricate molecular interactions and spatial constraints provide insight into the selectivity of PotD-PotABC for Spd over Put and Spm. Despite adding ATP to the sample before vitrification, no corresponding density indicative of ATP was observed in PotA.

Furthermore, we collected two datasets for the wild-type PotABC complex reconstituted into nanodiscs in the absence and presence of ATP, Mg^2+^, and Spd, respectively (fig. S4). For the latter, we obtained a map at approximately 3.5-Å resolution. However, no ligand was observed in the structure; perhaps because of flexibility, this further corroborates the necessity of the E to Q mutation (E173Q). The structure of the PotABC complex determined in nanodisc is virtually the same as that solved in detergent, with an RMSD of 0.60 Å for all 1240 pairs of Cα atoms (fig. S3E). This result certainly indicates that the PotABC E173Q mutant complex adopts the same structure as the wild-type complex.

### Structure of the Spd-bound PotD-PotABC in the pretranslocation state

**Fig. 2.**
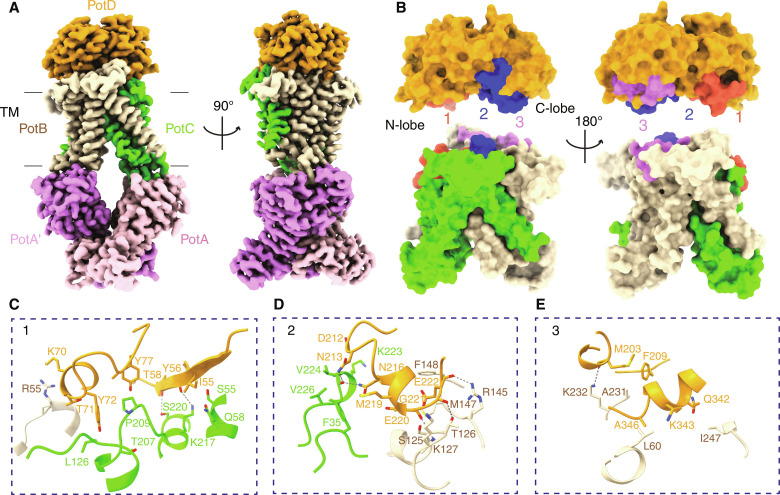
Structure of the PotD-PotABC complex in the pretranslocation state. (**A**) Overall architecture of the PotD-PotABC complex in the pretranslocation state. Two views from 90° angles are shown. The Spd-binding protein PotD is colored orange. (**B**) The interaction interfaces of PotD with the PotABC complex. For clarity, the figures showing the three interfaces on the PotD and PotABC complex are generated separately but with the same view. The three distinct interaction interfaces of PotD and PotBC are shown in red, blue, and purple, and labeled 1, 2, and 3, respectively. The N- and C-lobes of PotD (on the top) are indicated as well. (**C** to **E**) Detailed interactions of the three distinct interaction interfaces [from (B)] are shown with the interacting residues shown in sticks and labeled. The hydrogen bonds are shown as dashed lines.

PotD has been reported to specifically bind and deliver Spd to the PotABC transporter (*7*, *15*). We attempted to express the entire *potABCD* operon in *E. coli* with PotD protein tagged for affinity purification; however, only the PotD protein could be purified in this way. Thus, the PotD-PotABC complex was formed by incubating the purified PotD protein with the PotABC E173Q complex in the presence of Spd, ATP, and Mg^2+^. The formation of the entire PotD-PotABC complex was confirmed by size exclusion chromatography followed by SDS–polyacrylamide gel electrophoresis (SDS-PAGE) analysis (fig. S5A). The concentrated complex was vitrified for cryo-EM data collection and processing. The final map for the entire PotD-PotABC complex was reconstructed to 3.1 Å (Fig. 2A, figs. S5B and S6, and table S1).

The architecture of the entire PotD-PotABC complex reveals an inward-facing conformation, assigned as the pretranslocation state (Fig. 2A). The PotD-PotABC structure aligns well with the aforementioned apo-PotABC structure, with an RMSD of 0.33 Å for the 1108 pairs of Cα atoms (fig. S7A). PotD forms extensive interactions with both PotB and PotC along three distinct interfaces, with one (area 1) in the PotD N-lobe and the other two (areas 2 and 3) in the C-lobe (Fig. 2, B and E, and fig. S7B). Notably, only two pairs of hydrogen bonds are formed between the PotD N-lobe and PotB, namely, the main chain oxygen atom of Y56 and the hydroxyl group of T58 in PotD interact with the amine group of K217 and the hydroxyl group of S220 in PotB, respectively (Fig. 2C). In contrast, the interaction between the PotD C-lobe and PotBC is more extensive (Fig. 2, D and E). Namely, the residues D212 to E222 in PotD are sandwiched between PotC and PotB, establishing extensive contacts (Fig. 2D). In particular, the residues E220 to E222 in PotD interact with the residues K127, T126, and R145 in PotC through three pairs of hydrogen bonds. In addition, the PotD C-lobe forms hydrophobic interactions with L60, I247, and A231 in PotB, as well as one hydrogen-bonding interaction with K232 in PotB (Fig. 2E). To validate the relevance of the observed interactions between PotD and PotABC, we generated the PotD E220A/G221A/E222A triple mutant and assessed its binding to the PotABC E173Q. Compared to wild-type PotD, the PotD E220A/G221A/E222A mutant indeed binds to the PotABC E173Q rather weakly, given the very faint band of mutant PotD observed in the P1 fraction (equivalent to the intact complex PotD-PotABC) after size exclusion chromatography, suggesting a disruption of interaction caused by the mutagenesis of the interface residues (fig. S5, A and D). Together, the C-lobe of PotD plays a dominant role in interactions with the PotABC, thereby ensuring the stabilization of the entire complex. This asymmetric interaction indicates that the N-lobe of PotD could be more motile when delivering the Spd from PotD to the PotABC.

We observed one Spd molecule bound inside the PotD. The Spd is enclosed by the two lobes of PotD, similar to the isolated structure of Spd-bound PotD, with an RMSD of 0.75 Å for the 322 pairs of Cα atoms (fig. S7, A to C) (*27*). Notably, four acidic residues (E36, D168, E171, and D257) in PotD form the ionic interactions with the two amine groups in Spd, as well as four aromatic residues (W34, Y37, W229, and W255), which are in close contact with Spd via hydrophobic and van der Waals interactions (fig. S7C). However, we could not observe ATP binding on the basis of the lack of corresponding density. The structural similarity of the present Spd-bound PotD of the entire importer complex and the isolated PotD with Spd is not only in support of our structural modeling for Spd but also consistent with the previous extensive studies on the substrate specificity of PotD involving the aforementioned residues (*4*, *15*, *27*, *28*).

### Structure of ATP-bound PotD-PotABC in the translocation intermediate state

**Fig. 3.**
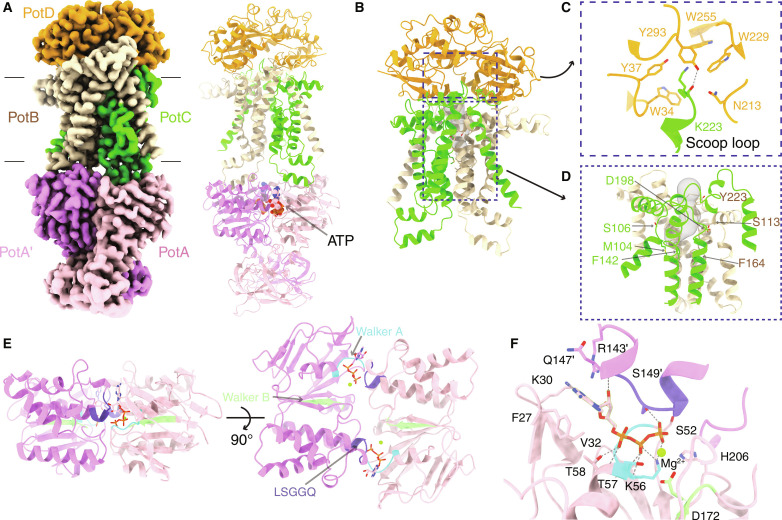
Structure of the PotD-PotABC complex in the translocation intermediate state. (**A**) Cryo-EM map of the PotD-PotABC complex in the translocation intermediate state (left). The corresponding atomic model is shown in cartoon (right), with the two bound ATP molecules shown as spheres. (**B**) The overall structure of PotD and PotBC in the translocation intermediate state. (**C**) A close-up view of the scoop loop (periplasmic loop 3) protruding into the Spd binding pocket. PotC K233 in the scoop loop directly interacts with the residues W34, Y37, N213, W229, W255, and Y293 in PotD. The hydrogen bond formed between K223 and Y293 is indicated by a dashed line. (**D**) A close-up view of the cavity formed by PotB and PotC. Following conformational changes in the PotD-PotABC complex upon ATP binding, a cavity (shown as gray surface) at the periplasmic side is formed between PotB and PotC. The residues implicated in Spd binding are labeled and shown as sticks. (**E**) The ATP binding sites in PotA. Two views from 90° angles are shown. The signature motifs Walker A, Walker B, and LSGGQ are indicated and colored cyan, pale green, and slate, respectively. ATP molecules are shown in sticks, with carbon, oxygen, nitrogen, and phosphate atoms colored pink, red, blue, and orange, respectively. The bound Mg^2+^ ions are shown as green spheres. (**F**) A close-up view of the ATP-binding pocket. ATP molecule is coordinated by residues from both PotA and PotA′ protomers. The residues involved in ATP binding are shown in sticks and labeled. The catalytic residue D172 is also shown. Hydrogen bonds are shown as gray dashed lines.

Through three-dimensional (3D) classification, we successfully captured the ATP-bound state of the PotD-PotABC complex. However, the preferred orientation issue of particles prompted us to revisit the sample preparation process and introduce the surfactant Fos-Choline-8 to the protein sample before vitrification. Eventually, we determined the structure of the PotD-PotABC complex in the ATP-bound state at 3-Å resolution (Fig. 3A, fig. S6, and table S1). This complex corresponds to the translocation intermediate state and sheds light on the dynamic molecular events underlying the transport of Spd.

In the translocation intermediate state, the overall interac-tion interface of PotD with PotB and PotC is similar to that of PotD-PotABC in the pretranslocation state. However, the detailed interactions have changed, including both newly established and disrupted interactions upon ATP binding (Fig. 2, B and E, and fig. S7D). The periplasmic loop P3 (residues 198 to 228, between TM5 and TM6; Fig. 1D) in PotC protrudes into the PotD binding pocket and is designated as the “scoop loop.” A similar feature is also observed in the maltose transporter MalFGK2 (in MalG) and the trehalose importer SugABC (in SugB) (*19*, *20*). The K223 residue in the scoop loop forms extensive contacts with the residues W34, Y37, N213, W229, W255, and Y293 in PotD that participate in Spd binding in the pretranslocation state (Fig. 3, B and C). In particular, the main chain oxygen atom of K223 forms one hydrogen bond with PotD Y293 (Fig. 3C). Given its chemical structure similarity to Spd, it is likely that K223 mimics Spd interaction with PotD residues and prevents Spd from rebinding to PotD, thereby facilitating its translocation to the cytosol. We also observed a large cavity (with a volume of around 200 Å^3^) formed inside the PotBC (Fig. 3D). However, we could not observe any Spd molecules bound inside this cavity. The residues (S113, F164, and Y223 in PotB; M104, S106, F142, and D198 in PotC) involved in Spd binding in the PotABC complex (Fig. 1H), are surrounding this cavity (Fig. 3D), suggesting that the Spd molecule likely undergoes progressive movement.

The NBDs of the two PotA protomers are packed tightly together to coordinate the binding of two ATP molecules at their interface, thereby resulting in the formation of a closed PotA homodimer (Fig. 3E and fig. S7E). The binding pockets of the two ATP molecules are symmetrically similar along the dimer interface, involving the signature Walker A and Walker B motifs from one PotA protomer and the LSGGQ motif from the other PotA protomer (PotA′) (Fig. 3, E and F). The residues S52, K56, T57, and T58 in the Walker A motif form extensive interactions with the triphosphate group of ATP, particularly T57, which also interacts with Mg^2+^. The residues H206 and S149′ (in the LSGGQ motif) from PotA′ interact and stabilize the gamma phosphate of ATP. The Q147 residue directly preceding the LSGGQ motif forms a hydrogen bond with the ribose of ATP. Last, the nucleobase adenine is sandwiched by PotA (F27 and K30) and PotA′ (R143′ and Q147′). The catalytic residue D172 in the Walker B motif interacts with the metal ion Mg^2+^ (Fig. 3F). To validate the ATP binding interface, we mutated PotA residues (F27 and T57 in the Walker A motif; R143; S149 in the LSGGQ motif; and the catalytic residue D172) to alanine, and the corresponding mutant PotABC complexes were purified, respectively (fig. S5C). Following the same ATPase assay method, we observed that all the mutations, except R143A, led to diminished ATPase activity (fig. S5E), suggesting that the aforementioned PotA residues are involved in either ATP binding or ATP hydrolysis. Particularly, mutating the catalytic residue D172 resulted in only marginal ATPase activity. Mutating the residue T57, which is in proximity to the catalytic center, also caused a substantial reduction in ATPase activity. The mutations of F27 and S149 sandwiching the ATP, showed 50% reduced activity compared to the native PotABC complex. R143 is likely a flexible residue only partially involved in ATP binding because its mutation to alanine had no strong effect on the ATPase activity. Collectively, the activity assays on these mutations nicely rationalize our structural data.

### Conformational changes from the pretranslocation to translocation intermediate state

**Fig. 4.**
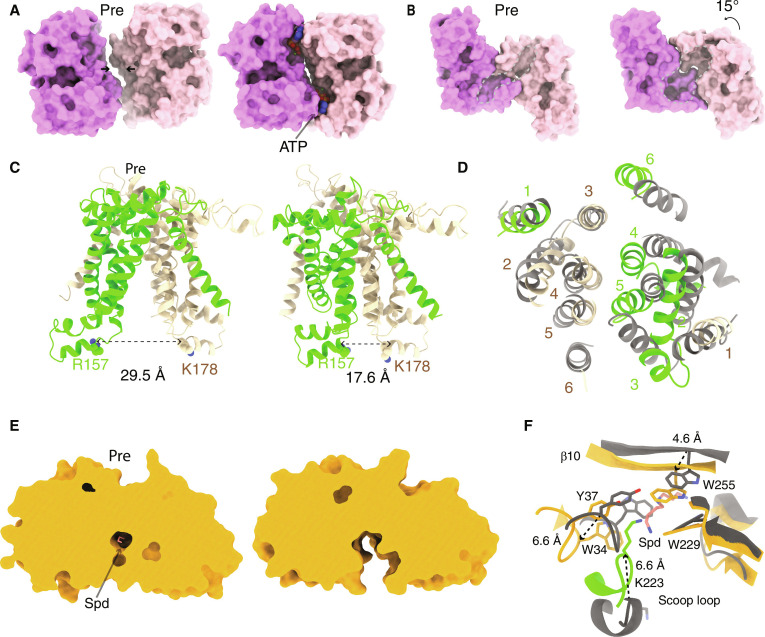
Conformational changes of the PotD-PotABC complex. (**A**) Structural comparison of PotD-PotABC in the translocation intermediate state (right) with that in the pretranslocation state (labeled as “Pre” and left) reveals changes. The NBDs move closer because of the ATP binding, with the shift direction indicated by black arrows. The NBDs are shown as surface representations when viewed from the periplasmic side. The two bound ATP molecules are shown as spheres. (**B**) Conformational changes of the regulation domain from the pretranslocation (Pre) state to the translocation intermediate state viewing from the cytosol side. The regulation domains are shown as surface representations. The gray dashed lines indicate the surface pockets. The rotation is also indicated. (**C**) The narrowing of the transmembrane pathway during transitioning from the Pre state (left) to the translocation intermediate state (right). The distances between the PotB K178 Cα atom and the PotC R157 Cα atom (with the two residues shown as spheres) in the two coupling helices are indicated. (**D**) Comparison of the orientation of PotB and PotC transmembrane helices (TMs) in the Pre state (gray) and translocation intermediate state (wheat and lime) viewed from the “top.” The TMs are numbered as in Fig. 1. (**E**) A cross-sectional surface view of the PotD from the PotD-PotABC complex in the Pre state (left) and translocation intermediate state (right). The PotD surface is clipped to reveal the Spd binding pocket and bound Spd. (**F**) The conformational changes in PotD from the Pre state (gray) to the translocation intermediate state (orange). PotD Spd binding residues and Spd are shown as sticks and labeled. The PotC scoop loop is shown in cartoon. The shifts of PotD W34, W255, and PotB K223 from the Pre state to the translocation intermediate state are indicated by the dashed arrows, with the distance indicated.

Compared to the pretranslocation state, extensive conformational changes are observed in the translocation intermediate state (Fig. 4). As depicted in Fig. 3, the two ATP molecules form bilateral interactions with the NBDs of PotA and PotA′ in the translocation intermediate state. Therefore, upon ATP binding, the two NBDs shift closer to one another (Fig. 4A). Following the movement of NBDs, a notable conformational change (~15° rotation relative to the twofold axis) in the regulation domain was also observed because of the ATP binding (Fig. 4B), and the distance between the two coupling helices changes from 29.5 (pretranslocation state) to 17.6 Å (translocation intermediate state) (Fig. 4C). This triggers a conformational change in PotB and PotC, leading to the complex transitioning from the inward-facing to the “outward-facing” state (Fig. 4C). Particularly, the transmembrane helices TM3 to TM6 of PotC are observed to shift toward PotB, forming a cavity for Spd to pass through (Fig. 4D).

Another remarkable difference between the two states is observed in PotD, more specifically, in its interaction interface with the PotBC heterodimer. In the pretranslocation state, Spd is enclosed by the N- and C-lobes of PotD in a tightly “closed” form. In contrast, the two lobes of PotD are observed to be in an “open” form in the translocation intermediate state, resulting in the formation of a cleft wide enough to provide a direct pathway for the Spd translocation (Fig. 4E). In particular, the opening of the Spd binding pocket involving the scoop loop in PotC is of particular interest. Compared with Spd-bound PotD in the pretranslocation state, the β10, one of the two hinges connecting the N- and C-lobes and similar to a “lid,” is shifted by approximately 4.6 Å toward Spd, resulting in the structural clash and displacement of Spd by the residue W255 (Fig. 4F). Note that W255 is critical for Spd binding; its mutation results in a markedly decreased affinity of PotD for Spd (*15*). Concurrently, the loops (with W34 in the N-lobe and W229 in the C-lope) move apart (i.e., W34 in the loop is displaced by 6.6 Å) to open up the Spd binding pocket for its translocation. Furthermore, the scoop loop P3 shifts up to 6.6 Å and projects into the Spd binding site (Fig. 4F). Notably, the conformational change of the scoop loop orients the tip of the residue K223 to partially overlap with the Spd position in PotD of the pretranslocation complex.

### The Spd translocation pathway and a proposed mechanism of the PotD-PotABC transporter

**Fig. 5.**
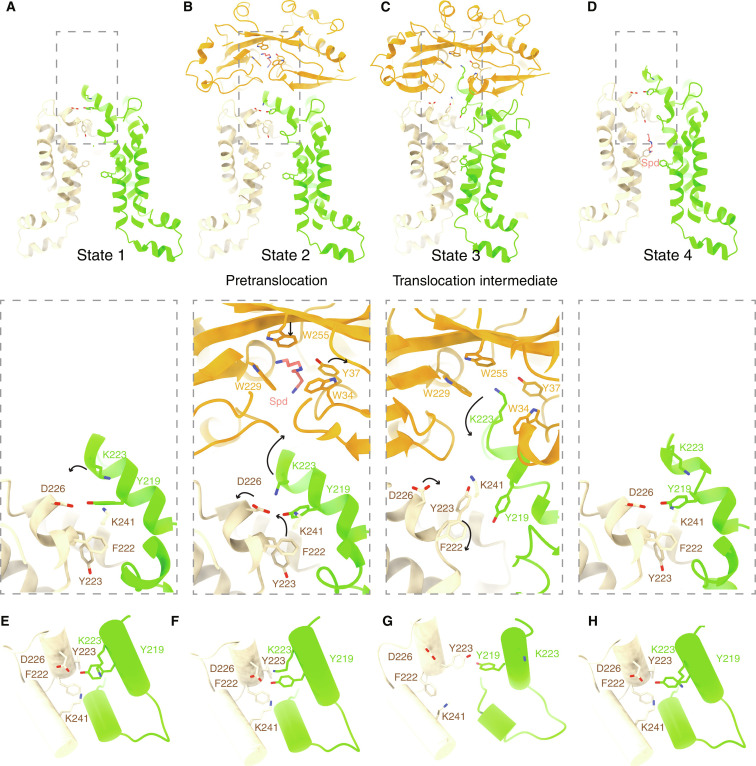
The conformational changes in the Spd translocation pathway. (**A** to **D**) Structural comparison of the PotBCD interaction interfaces of the four states along the Spd translocation pathway reveals significant conformational changes. The four states are as follows: state 1, apo PotABC; state 2, PotD-PotABC pretranslocation state; state 3, PotD-PotABC translocation intermediate state; state 4, Spd-bound PotABC. The periplasmic gating residues, the cytosolic side gating residues, and the Spd-interacting residues in PotD are shown in sticks and labeled. Close-up views of the gating residues on the periplasmic side are shown below. The conformational changes of the key residues compared with those in the following state are indicated by black arrows. (**E** to **H**) Top view of the periplasmic gates in the four states. The gating residues are shown as sticks. The TMs are shown as tubes.

In the apo conformation of the PotABC complex (state 1, Fig. 5A), the PotB (D226 and K241) and PotC (Y219) form the plane interaction network to enclose the PotABC translocation pathway at the periplasmic side. The Spd-bound PotD in a closed form interacts with the PotABC complex to form the entire PotD-PotABC complex in the pretranslocation state (state 2, Fig. 5B). The scoop loop of PotC shifts toward PotB because of its newly established interaction with the D212 region in the PotD C-lobe (Fig. 2D). Therefore, a new interaction network is formed, involving PotB (F222, D226, and K241) and PotC (K223 and Y219). The residue Y223 in PotB adopts an inward facing conformation. In the translocation intermediate state (state 3, Fig. 5C), ATP binding to the PotA NBDs induces dimerization of NBDs and conformational changes of the coupling helices as well as the transmembrane helices (particularly TM3 to TM5), resulting in an outward-facing state of PotBC (Fig. 5C). Spd is displaced by W255 in PotD, and the scoop loop inserts into the cleft formed by the N- and C-lobes of PotD, with K223 occupying part of the Spd binding site. In addition, the interaction network in PotB (F222, D226, and K241) and PotC (Y219 and K223) is disrupted, and its site is partially occupied by the side chain of Y223 (the outward-facing conformation) in PotB. Consequently, the entrance of the transmembrane pathway widens, and a cavity is formed to accommodate Spd. In the next state (state 4, Fig. 5D), Spd has been transported to the internal cavity, as seen in Fig. 1H. The residues above are found to return to the same conformation as that in state 1, resulting in a closed gate at the periplasmic side (inward-facing conformation). In particular, the residue Y223 in PotB faces inward to form a hydrogen-bonding interaction with the Spd terminal amide group (Fig. 1H). As the translocation pathway opens further on the cytoplasmic side, the residues (PotB F164 and L213; and PotC F142 and L191) move apart, allowing Spd to be released to the cytosol.

We hypothesized that the PotD-stimulated PotABC ATPase activity would be affected by the altered translocation pathway of Spd in the PotABC through mutation of those residues important for translocation. To validate the importance of the gating residues and the scoop loop for Spd transportation, we mutated PotB residues (Y223 and D226) in the periplasmic gate and PotC residue K223 in the scoop loop to alanine. We tested the ATPase activity of all the mutants in the presence and absence of PotD and observed that PotD had no notable effect on the ATPase activity of the PotB (Y223A and D226A) and PotC (K223A) mutants, which is different from that for the wild-type PotABC complex (fig. S5F). This abolished PotD stimulation on PotABC ATPase activity likely suggests that these residues are indeed vital for Spd translocation.

**Fig. 6.**
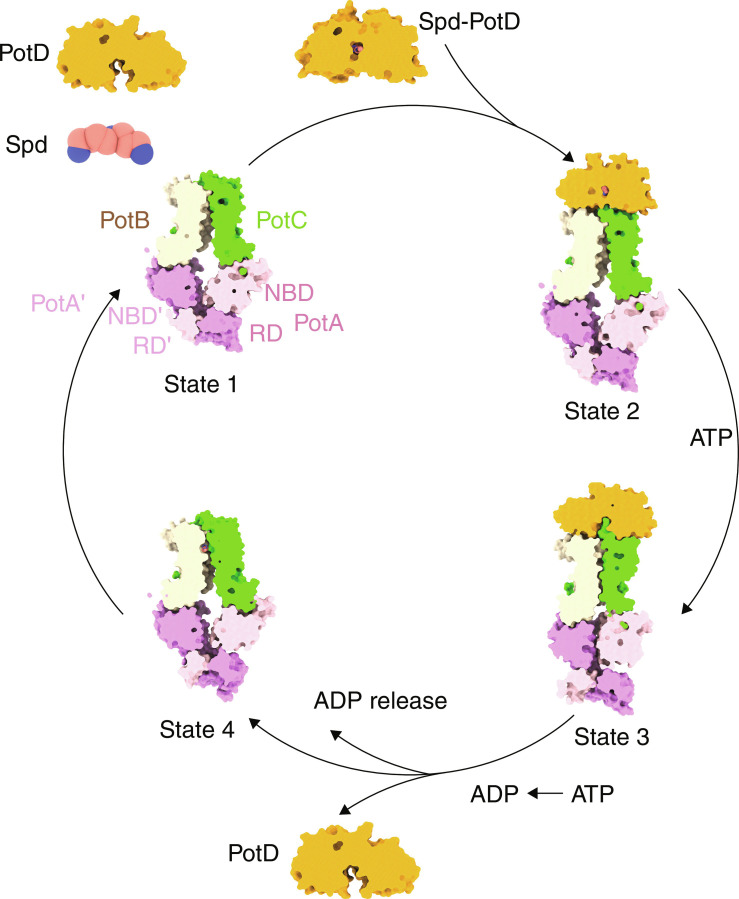
Proposed Spd uptake mechanism of the PotD-PotABC transporter. PotD binds free Spd and subsequently associates with the transmembrane PotABC complex (state 1), thus forming the Spd-bound PotD-PotABC complex (state 2). Two ATP molecules bind to the NBDs of PotA and PotA′ protomers, thereby inducing conformational changes in the PotABC translocation pathway (state 3). The PotB Y223 moves from the inward-facing conformation to the outward-facing conformation, along with the opening of the periplasmic gate formed by residues in PotB (F222, D226, and K241) and PotC (Y219 and K223). The scoop loop of PotC protrudes into the Spd binding pocket. Spd is then released to the transmembrane passage. ATP hydrolysis provides the energy for switching from the outward-facing conformation to the inward conformation (state 4). PotD, phosphate, and adenosine diphosphate (ADP) dissociate from the PotABC complex. Last, the Spd is released into the cytosol.

Together, our study reveals a series of states delineating the Spd translocation pathway at atomic resolution (Fig. 5, A to H), allowing us to propose the following mechanism (Fig. 6). When bacterial cells sense an increasing demand for Spd, the Spd carrier PotD binds free Spd in the periplasm and encloses the Spd in a closed state. Subsequently, the Spd-bound PotD binds to the “inward-facing state” PotABC transporter in the plasma membrane. ATP binding to the PotA triggers a series of conformational changes in the entire PotD-PotABC transporter, leading to the formation of the outward-facing state. The conformational changes cause the opening of the PotD and the insertion of the scoop loop into the cleft of the PotD. Consequently, Spd is translocated into the outward-facing cavity. Next, ATP hydrolysis likely induces conformational changes of the two NBDs of PotA and dissociation of PotD from the PotABC, as well as opening toward the cytoplasmic side (the inward-facing state). At the same time, the interaction network on the periplasmic side is re-established, and the gate is closed. Possibly in association with adenosine diphosphate or phosphate (Pi) release, further opening toward the cytoplasmic side allows the Spd to be released to the cytosol.

## DISCUSSION

Polyamines are polycationic molecules ubiquitously found in all kingdoms of life, where they perform essential functions in diverse cellular processes. We determined the structures of the Spd-preferential uptake complex PotABC from *E. coli* in both apo- and Spd-bound forms. Furthermore, we were able to determine the PotD-PotABC complex in the pretranslocation state and the translocation intermediate state. Superposing the structure of Spd-bound PotD from the PotD-PotABC complex to the crystal structure of Spd-bound PotD reveals a high similarity (fig. S7, A and C) (*27*). The structural comparison of the PotD of PotD-PotABC in the pretranslocation state and the translocation intermediate state reveals the dynamic and flexible features of PotD two lobes and that the connecting hinge β10 is crucial for binding and delivering Spd. Our structure of PotD-PotABC with ATP bound to PotA in the translocation intermediate state demonstrates that the LSGGQ and Walker A motifs from the two PotA protomers play a key role in coordinating ATP, which is consistent with the previous reports on ABC transporters (*19*, *20*, *29*). Consistent with our structural analysis, our mutagenesis studies validated the residues important for ATP binding, Spd trans-location, and PotD interaction with PotABC by using ATPase activity and gel filtration assays (fig. S5, C to F). The overall PotD-PotABC structures are similar to the previously reported type 1 ABC importers and exhibit a 15° rotation of the regulation domain from the pretranslocation state to the translocation intermediate state (figs. S4, A and B, and S8, A and B). The most notable structural divergence lies in the substrate-binding protein, which is not unexpected given its role in ensuring substrate specificity. As substrate-binding protein-dependent ABC transporters, it is plausible that these importers use a similar transportation mechanism, where the carrier protein delivers the substrate to the importer for uptake.

*E. coli* uses two different uptake systems for polyamines: PotD-PotABC for Spd and PotF-PotGHI for Put (*14*). Previous structural studies of the isolated carrier proteins PotD and PotF revealed that these two proteins specifically recognize their preferred substrate, resulting in the preferential uptake of the corresponding polyamines, Spd and Put, respectively (*4*, *27*, *28*, *30*). In addition to the substrate specificity of PotD, the various structures reported here, particularly the pretranslocation and the translocation intermediate states, offer atomic insights into how Spd is imported along the translocation pathway, suggesting that the translocation pathway also confers the substrate selectivity. At the periplasmic side of the translocation pathway, a set of residues (F222, D226, and K241 in Pot B and K223 in PotC) and PotC (Y219 and K223) form a network of interaction to act as a “gatekeeper” (Fig. 5, E to H). This gatekeeper regulates access to the pathway, controlling the passage of Spd molecules. In addition, the neighboring residue Y223 in PotB alternates between outward- and inward-facing conformations, thereby possibly directly facilitating Spd translocation. In addition, the PotB residues S113, I160, F164, L213, and Y223 as well as the PotC residues M104, S106, and D198 interact with Spd located in the transmembrane pathway during translocation (Fig. 1H). The synergistic action of these two sites, the gatekeeper region and the Spd-interacting residues in the transmembrane translocation pathway, collectively enhance the specificity and selectivity of polyamine transport. The ATPase activity of PotABC is significantly reduced by adding Spd (fig. S1E), which is consistent with a previous report (*24*). In line with this notion, the Spd uptake activity has been reported to be down-regulated after *E. coli* cells were cultured in the presence of polyamines (*31*). The extensively negatively charged regulation domain of PotA possibly enables the specific or nonspecific binding of the Spd (fig. S8C). The increased cytosolic concentration of polyamines (polycations) could lead to their binding to the regulation domain, thus locking the PotABC transporter in the inhibited state to ensure polyamine homeostasis. However, this requires further investigation.

In contrast to the prokaryotic polyamine uptake system, which comprises five subunits consisting of four different proteins, the human polyamine transporter ATP13A2 (PARK9) presents a distinct structure as a single P5-type ATPase localized on the lysosome membrane (*17*). This structural diversity underscores fundamental differences in the overall architecture and translocation mechanism between ATP13A2 and PotD-PotABC (fig. S8D) (*2*, *17*). Sequence alignment of the Spd importing system PotD-PotABC from *E. coli*, *Bacillus subtilis*, *Mycobacterium tuberculosis*, *Streptomyces scabiei*, *Cyanobacteria bacterium*, and *Pseudomonas aeruginosa* reveals conservation of the PotA ATP binding motifs and PotD Spd binding residues, suggesting similar recognition and translocation mechanisms across bacteria (figs. S9 to S12) (*32*, *33*). Furthermore, it is noteworthy that polyamines play crucial roles in the proliferation and growth of human pathogens. For instance, polyamines are implicated in biofilm formation in *Vibrio cholerae*, *P. aeruginosa*, and *Yersinia pestis* (*23*–*25*), as well as in capsular polysaccharide deposition in *Streptococcus pneumoniae* (*26*, *27*). Given the significance of polyamines in pathogenicity, disrupting polyamine homeostasis presents a promising approach for the development of therapeutic strategies for treating diseases caused by these pathogens.

## MATERIALS AND METHODS

### Cloning

*E. coli* K12 strain operon *potabcd* was cloned into the pET28a derivative vector pOPTChis with a C-terminal noncleavable hexahistidine tag. The plasmid pOPTChis-PotABC was generated by deleting the PotD from the plasmid pOPTChis-PotD-PotABC. The PotA (F27A, T57A, R143A, S149A, D172A, and E173A), PotB (Y223A and D223A), PotC (K223A), and PotD (W34A, W229A, and E220A/G221A/E222A) mutants were generated by site-directed mutagenesis. The constructs were verified by DNA sequencing.

### Protein expression and purification

The PotABC wild-type, PotA (F27A, T57A, R143A, S149A, D172A, and E173Q), PotB (Y223A and D223A), and PotC (K223A) variants of the PotABC complex were purified similarly. Briefly, the plasmid was transformed into *E. coli* C41 cells for protein expression. A single colony was selected and cultured in 2xYT media. Protein expression was induced by 0.2 mM isopropyl-β-d-thiogalactopyranoside (IPTG), and cells were grown at 20°C overnight. Cells were harvested by centrifugation (4,000*g* for 10 min) and resuspended in buffer [20 mM tris-HCl (pH 7.5) and 150 mM NaCl]. Bacteria were lysed by sonication and centrifuged at 10,000*g* for 10 min to remove the cell debris. The supernatant was loaded for ultracentrifugation at 150,000*g* for 1 hour. The membrane was resuspended in buffer [20 mM tris-HCl (pH 7.5), 150 mM NaCl, 10% glycerol, and 1% n-Dodecyl β-D-maltoside (DDM)] and incubated at 4°C for 1 hour. Then, the solubilized membrane proteins were collected via centrifugation at 150,000*g* for 1 hour. The supernatant was collected and incubated with pre-equilibrated nickel–nitrilotriacetic acid (NTA) beads at 4°C for 1 hour on an end-over-end shaker. The protein complex was purified by gravity flow chromatography by extensive washing and subsequent elution with 20 mM tris-HCl (pH 7.5), 150 mM NaCl, 0.01% Lauryl Maltose Neopentyl Glycol (LMNG), and 200 mM imidazole. The PotABC complex was concentrated using a 100-kDa molecular weight cutoff (MWCO) concentrator (Millipore) and loaded onto a Superose 6 10/30 gel filtration column (Cytiva) pre-equilibrated with buffer [20 mM tris-HCl (pH 7.5), 150 mM NaCl, and 0.001% LMNG]. The fractions containing the target proteins were pooled, concentrated to 10 mg/ml, and snap frozen.

The PoD wild-type and PotD (E220A/G221A/E222A) mutants were purified by expressing the PotD-PotABC protein complex with the C-terminal hexahistidine tag on PotD in *E. coli* C41 cells. After transformation, a single colony was selected and cultured in 2xYT media. Protein expression was induced by 0.2 mM IPTG, and cells were grown at 20°C overnight. Cells were harvested by centrifugation (4000*g* for 10 min) and resuspended in buffer [20 mM tris-HCl (pH 7.5) and 150 mM NaCl]. Bacteria were lysed by sonication and centrifuged at 60,000*g* for 1 hour to remove the cell debris. The supernatant was collected and incubated with pre-equilibrated nickel-NTA beads at 4°C for 1 hour on an end-over-end shaker. The PotD was purified by gravity flow chromatography. After extensive washing and subsequent elution with 20 mM tris-HCl (pH 7.5), 150 mM NaCl, and 200 mM imidazole, the PotD was concentrated using a 10-kDa MWCO concentrator (Millipore) and loaded onto a Superose 6 10/30 gel filtration column (Cytiva) pre-equilibrated with buffer [20 mM tris-HCl (pH 7.5) and 150 mM NaCl]. The fractions containing the target proteins were pooled, concentrated to 10 mg/ml, and snap frozen.

For the PotD-PotABC E173Q protein complex preparation, the PotABC E173Q protein was incubated with PotD in the presence of 2 mM ATP, Mg^2+^, and Spd at 4°C for 1 hour. After incubation, it was loaded to the Superose 6 10/30 gel filtration column (Cytiva) pre-equilibrated with buffer [20 mM tris-HCl (pH 7.5), 150 mM NaCl, and 0.001% LMNG]. The PotD-PotABC E173Q complex peak was confirmed by SDS-PAGE. The peak fractions were concentrated to 10 mg/ml and snap frozen. The interaction test between PotD mutant E220A/G221A/E222A and PotABC E173Q was done similarly.

### Nanodisc reconstitution

The MSP1E3D1 purification and nanodisc reconstitution in general followed the previous method (*34*). Briefly, asolectin in chloroform (Avanti, USA) was dried in a nitrogen stream and resuspended in buffer [20 mM Hepes-NaOH (pH 7.5) and 150 mM NaCl]. To assemble PotABC into nanodiscs, a ratio of 1:3:200:1000 (PotABC: MSP1E3D1:lipid:DDM) was used. The mixture was incubated for 30 min at 4°C. After, 0.1 mg/ml of prewashed Bio-Beads (Bio-Rad) was added and incubated overnight at 4°C. The sample was loaded to a Superose 6 column equilibrated with 20 mM tris-HCl (pH 7.5) and 150 mM NaCl. Fractions containing the PotABC nanodisc were collected for cryo-EM study.

### ATPase assay

The ATPase assay was performed using a commercial ATPase kit (Sigma-Aldrich, MAK113) according to the manufacturer’s protocol with minor modifications. Briefly, a final concentration of 1 μM of the PotABC complex (wild-type or mutant) was incubated in a reaction buffer [25 mM tris-HCl (pH 7.5), 150 mM NaCl, and 0.001% LMNG] at 37°C for 10 min with 1 mM ATP and 1 mM Mg^2+^. For the Spd inhibition test and PotD activation test, Spd was used at a concentration of 1 mM, and PotD was used at a concentration of 5 μM. The components were added to the individual wells of the 96-well plates. The final reaction volume in each well was 20 μl. After incubation, 100 μl of malachite green reagent was added to each reaction well and incubated on the shaker for 30 min. The absorbance at 620 nm was measured using the plate reader (Tecan). The standard curve was determined by preparing a serial dilution of phosphate standard in water with concentrations of 20, 10, 5, 2.5, 1.25, and 0.6 μM in each well. The phosphate products were calculated on the basis of the standard curve. The experiments were performed in triplicate.

### Cryo-EM data collection and processing of PotABC in detergent

For both the PotABC E173Q complex and the PotD-PotABC E173Q complex dataset 1, a total of 3.5 μl of protein sample at a concentration of 10 mg/ml was applied to the Quantifoil Cu R1.2/1.3 grids. To address the preferred orientation issue of the ATP-bound state of the PotD-PotABC E173Q complex, the sample was added with a final concentration of 3 mM Fos-Choline-8 before vitrification, and Quantifoil Au R1.2/1.3 was used for dataset 2 collection. The datasets were collected on a Titan Krios (Thermo Fisher Scientific, USA) 300-kV electron microscope equipped with a GIF Quantum energy filter and a Falcon4i detector with a defocus range between −0.6 and −1.2 μm. The magnification was ×165k with a pixel size of 0.76 Å.

Micrographs were imported to Cryosparc4.1 for patch motion correction and patch CTF estimation (*35*). Micrographs with an estimated CTF resolution higher than 6 Å were selected for subsequent processing. Particles were auto-picked by the blob picker, manually curated, and extracted with box size 80 and pixel size 3.04 Å. All the extracted particles were subjected to a reference-free 2D classification. Good classes with clear protein features were selected and used for initial model building and heterogeneous refinement. Only the reasonable models were selected, and the good particles were re-extracted to box size 200 and pixel size 1.216 Å. The re-extracted particles were used for heterogeneous refinement and, subsequently, nonuniform refinement. The resulting maps for apo PotABC, Spd-PotABC, Spd-PotD-PotABC, and ATP-PotD-PotABCD reached 3.1, 3.1, 3.1, and 3 Å based on gold-standard Fourier shell correlation (FSC) = 0.143, respectively. The reconstructed EM maps were density modified by DeepEMhancer for illustration purposes (*36*). The crystal structure of PotD [Protein Data Bank (PDB) ID: 1POT], AlphaFold 2–predicted models of PotA, PotB, and PotC (AlphaFold database accession codes: AF-P69874-F1, AF-P0AFK4-F1, and AF-P0AFK6-F1) were used for model building, which was manually adjusted with COOT, and real space refinement in Phenix Suite (*37*–*39*). All the structure-related figures were prepared by ChimeraX (*40*).

### Cryo-EM data collection and processing of the wild-type PotABC in nanodisc

For the nanodisc-reconstituted PotABC wild-type protein sample, 3.5 μl of the protein alone or with the addition of 1 mM Mg^2+^ and ATP at a concentration of 0.8 mg/ml was applied to a graphene-coated Quantifoil Au R1.2/1.3 grid. The two datasets were collected on a Titan Krios (Thermo Fisher Scientific, USA) 300-kV electron microscope equipped with a GIF Quantum energy filter and a Falcon4i detector with a defocus range between −0.6 and −1 μm. The magnification was ×130k with a pixel size of 0.97 Å.

Micrographs were imported to Cryosparc4.1 for patch motion correction and patch CTF estimation (*35*). Micrographs with an estimated CTF resolution higher than 4 Å were selected for subsequent processing. Particles were picked template based, extracted, and subjected to a reference-free 2D classification. Good classes with clear protein features were selected and used for initial model building and heterogeneous refinement. Only the reasonable models were selected, and the good particles were re-extracted to box size 200 without binning. The re-extracted particles were used for heterogeneous refinement and, subsequently, nonuniform refinement. Unfortunately, no high-resolution map can be obtained from the apo PotABC nanodisc sample. However, in the presence of Mg and Spd, one 3.5-Å map can be obtained on the basis of gold-standard FSC = 0.143. The reconstructed EM map was density modified by DeepEMhancer for illustration purposes (*36*). The previous PotABC E173Q model was used for rigid body fitting to the map. The model was manually adjusted with COOT and real-space refinement in Phenix Suite (*37*–*39*). The structure-related figures were prepared by ChimeraX (*40*).
