# Supplementary material for: Structural insights into polyamine spermidine uptake by the ABC transporter PotD-PotABC
Source: Sci Adv. 2024 Sep 20;10(38):eado8107. doi: 10.1126/sciadv.ado8107 (PMC11414716; doi:10.1126/sciadv.ado8107)
Supplement: Supplementary file 1 — Figs. S1 to S12 Table S1 [file sciadv.ado8107_sm.pdf]

Science Advances
AAAS

Supplementary Materials for

Structural insights into polyamine spermidine uptake by the ABC transporter PotD-PotABC

Zhu Qiao *et al.*

Corresponding author: Yong-Gui Gao, ygao@ntu.edu.sg

Sci. Adv. **10**, eado8107 (2024)
DOI: 10.1126/sciadv.ado8107

This PDF file includes:

Figs. S1 to S12
Table S1

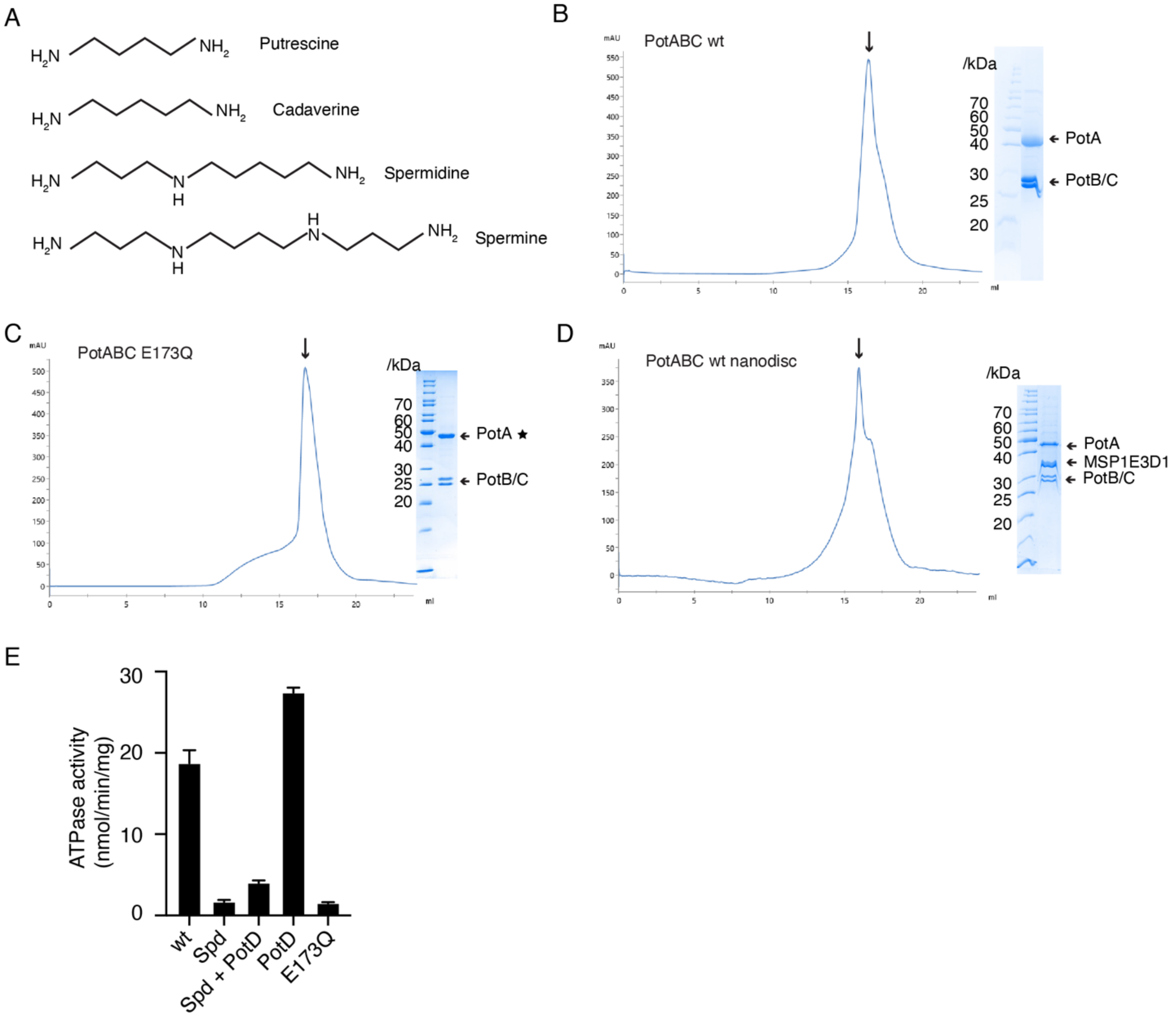

Fig. S1. Purification and ATPase activity assay of the spermidine-preferring PotABC complexes.

A. Chemical structures of the polyamines putrescine (Put), cadaverine (Cad), spermidine (Spd), and spermine (Spm). B. Size-exclusion chromatography elution profile of the wild-type (wt) PotABC complex (left) and its SDS-PAGE analysis (right). The arrow indicates the complex elution peak, and the final sample quality is analyzed by SDS-PAGE. C. Size-exclusion chromatography elution profile of the mutant PotABC E173Q complex (left) and its SDS-PAGE analysis (right). The arrow indicates the complex elution peak. The final sample quality is checked by SDS-PAGE, and the star indicates that the corresponding band has been confirmed by mass-spectrometry analysis. D. Size-exclusion chromatography elution profile of the nanodisc-reconstituted wild-type PotABC (left) and the SDS-PAGE (right). The final sample quality is assessed by SDS-PAGE, and the membrane scaffold protein MSP1E3D1 (used for nanodisc reconstitution) is also indicated. E. ATPase activity assay of PotABC in detergent. The ATPase activity of wt PotABC (alone or treated with 1 mM Spd, 1 mM Spd, and 5 µM PotD, or 5 µM PotD, respectively) and the mutant PotABC E173Q are shown. The ATP hydrolysis activity is

shown as the amount (nmol) of phosphate released by 1 mg of protein in 1 minute. The quantity of phosphate was calculated based on the standard curve. Three independent experiments were carried out, and the mean values with standard deviations are shown.

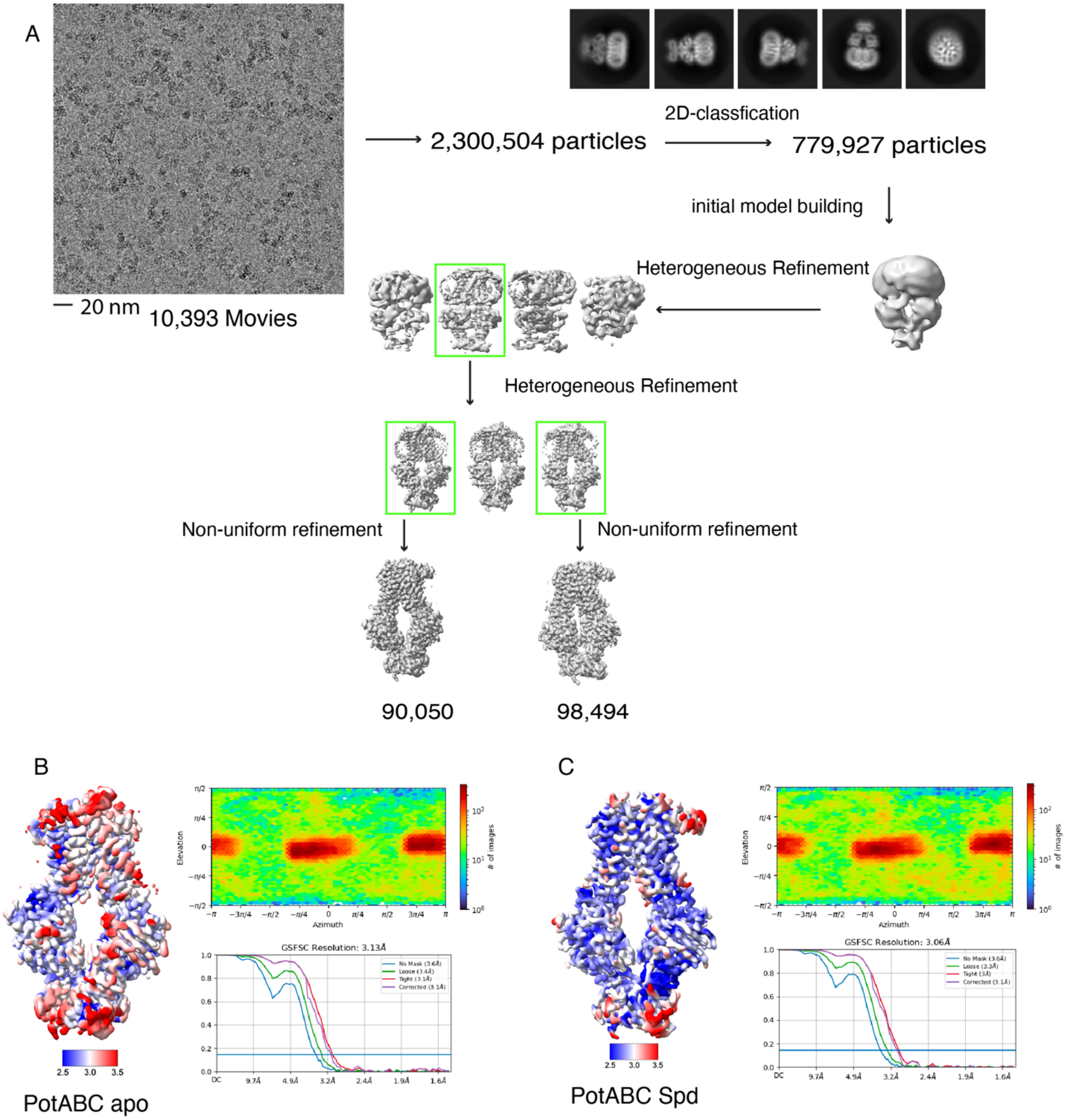

Fig. S2. Cryo-EM data processing of the PotABC E173Q dataset.

A. The workflow of the PotABC E173Q dataset processing. A representative of raw micrographs and 2D class averages are shown. The scale bar is 20 nm. The number of particles is given. B. Local resolution estimation of the apo PotABC electron density map. The angular distribution and the final FSC curve are shown on the right. C. Local resolution estimation of the Spd-bound PotABC electron density map. The angular distribution and the final FSC curve are shown on the right.

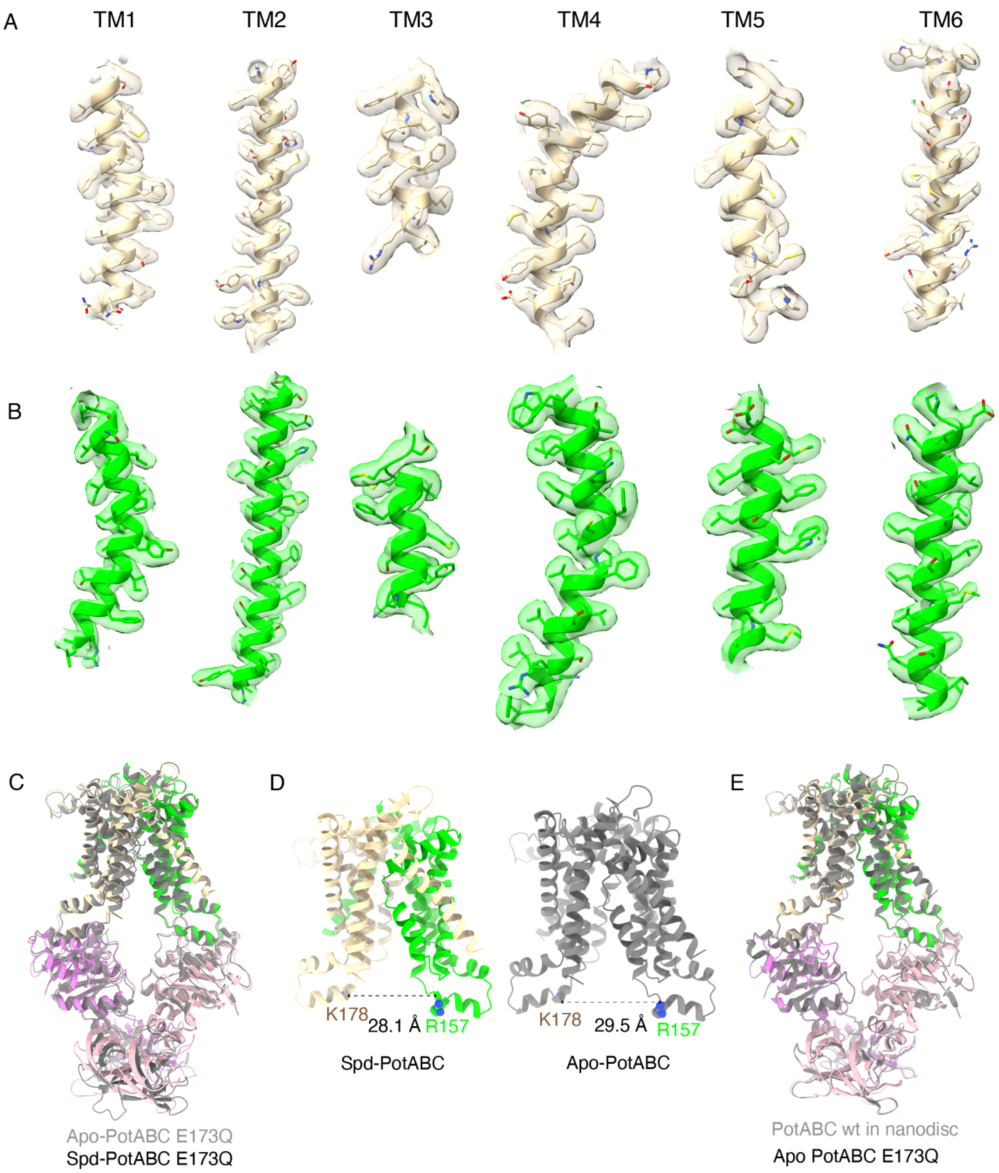

Fig. S3. The model fitting and structural comparison of PotABC.

A. The model fitting of the six transmembrane helices of PotB to the apo PotABC map. B. The model fitting of the six transmembrane helices of PotC to the apo PotABC map. C. Structural comparison of the apo form PotABC E173Q (gray) to the Spd-bound PotABC E173Q. D. The slight closing of the transmembrane channel of PotBC in the Spd-bound PotABC E173Q structure compared to the apo form. The distances between the two coupling helices (between the PotB K178 Cα atom and the PotC R157 Cα atom) are shown. E. Structural comparison of the apo form wild-type PotABC in nanodisc (gray) to the apo form PotABC E173Q in detergent.

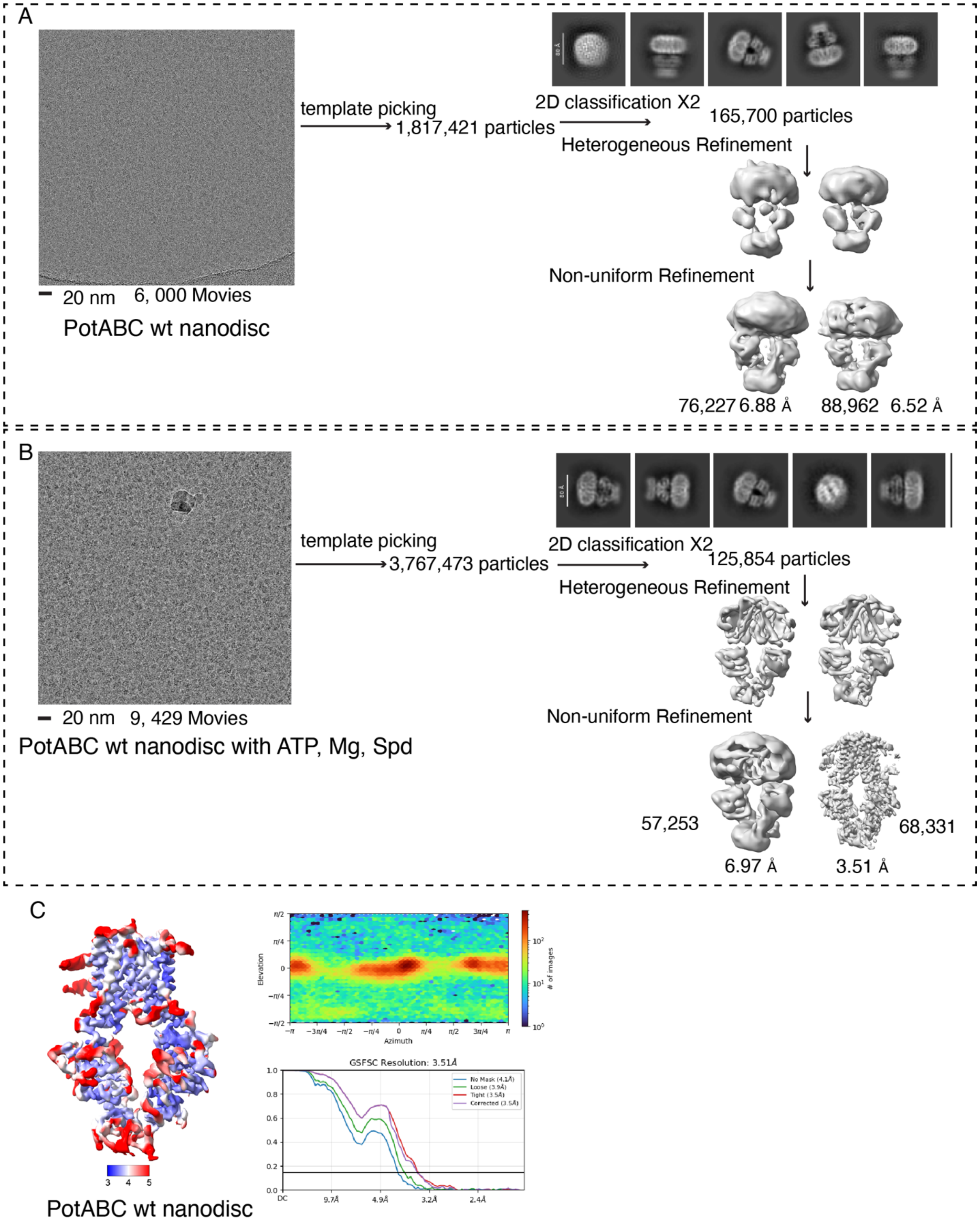

Fig. S4. Cryo-EM data processing of the wild-type PotABC in nanodisc.

A. The workflow of the wild-type PotABC in nanodisc dataset 1 (without ligands) processing. A representative raw micrograph and the 2D class averages are shown. The scale bar is 20 nm. No

high-resolution reconstruction can be obtained from this dataset. B. The workflow of the wild-type PotABC in nanodisc dataset 2 (with addition of ATP, Mg^{2+}, and Spd) processing. One high-resolution model of PotABC complex is obtained with no ligand bound. C. Local resolution estimation of the map (from panel B) of wild-type PotABC complex in nanodisc. The angular distribution and the final FSC curve are shown on the right.

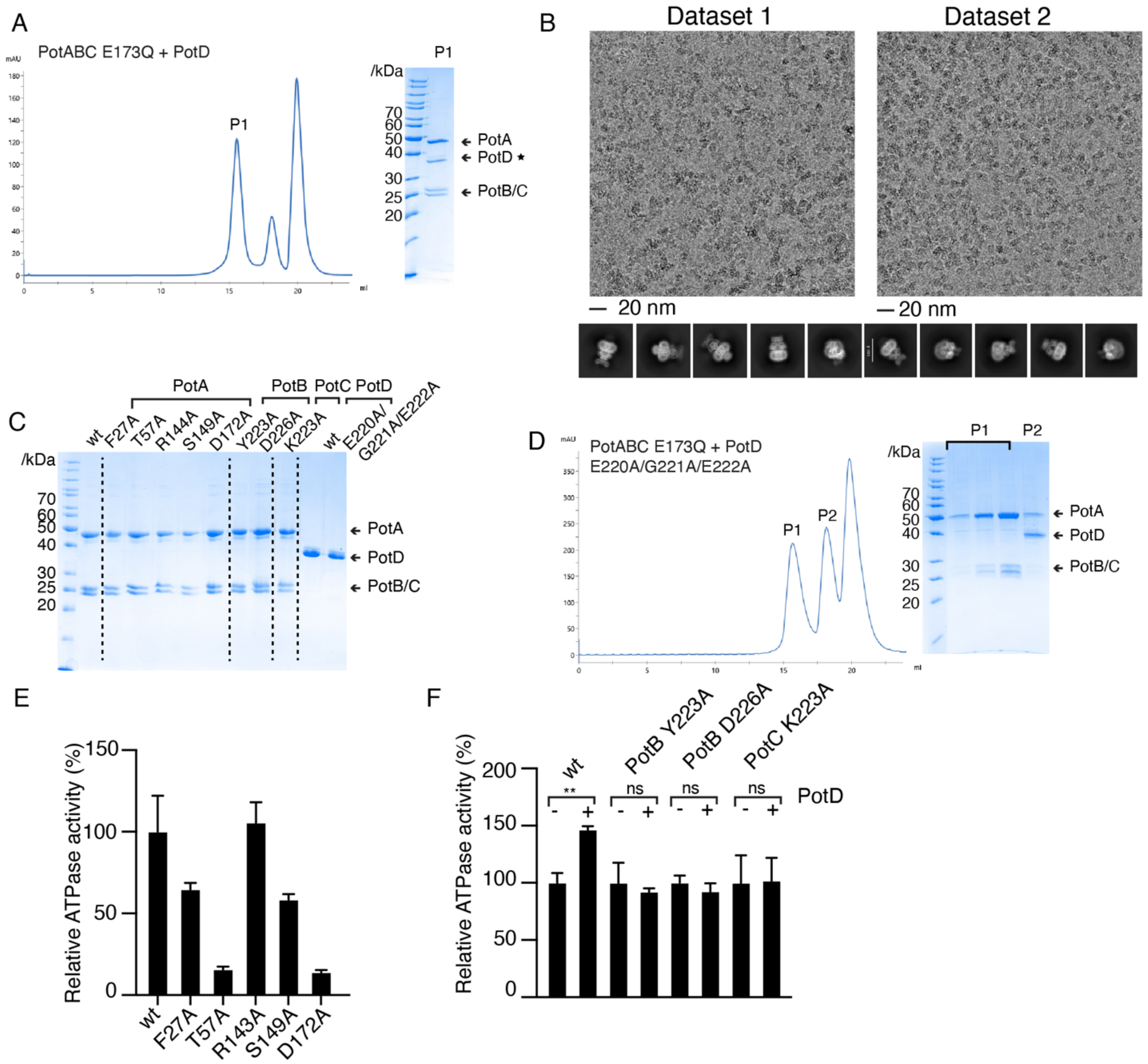

Fig. S5. PotD-PotABC complex formation, cryo-EM analysis, mutagenesis, and biochemcial assays.

A. Size-exclusion chromatography elution profile (left) of the *in vitro* formed PotD-PotABC E173Q complex. P1 indicates the peak corresponding to the target entire complex in the elution profile. The final sample quality (P1) is analyzed by SDS-PAGE (right). The star indicates that the PotD band is confirmed by mass-spectrometry analysis. B. The representative raw micrographs (top) and 2D class averages (bottom) of PotD-PotABC E173Q dataset 1 and dataset 2. The scale bar for micrographs is 20 nm. C. The SDS-PAGE analysis of purified wild-type (wt) PotABC complex and its subunits (PotA, PotB, PotC) mutants, as well as wt PotD and its mutant. D. Size-exclusion chromatography elution profile (left) of PotD E220A/G221A/E222A mutant binding to the PotABC E173Q. The SDS-PAGE analysis of fractions corresponding to elution peaks (P1 and P2) is shown on the right. Compared to wt PotD in the panel A, only a very faint band of PotD E220A/G221A/E222A mutant could be observed in P1 fractions. The P2 peak indicates the excess PotD mutant. E. ATPase activity assay of wt and mutant PotABC complexes. The wild-type

PotABC activity is shown as 100%, and the relative ATPase activities of PotABC complexes containing PotA mutants are shown. Three independent experiments were carried out and the mean values with standard deviations are presented. F. The effect of PotD on the ATPase activity of PotABC mutants. The ATPase activity of wt PotABC and complexes containing mutant PotB (Y223A and D226A) or mutant PotC (K223A) with and without PotD are shown. PotD stimulates the wt PotABC complex only. Three independent experiments were carried out and the mean values with standard deviations are shown. The statistical analysis is done by unpaired two-tailed student *t*-test with $t\ (2) = 8.73$ and $p = 0.0056$. ns: not significant.

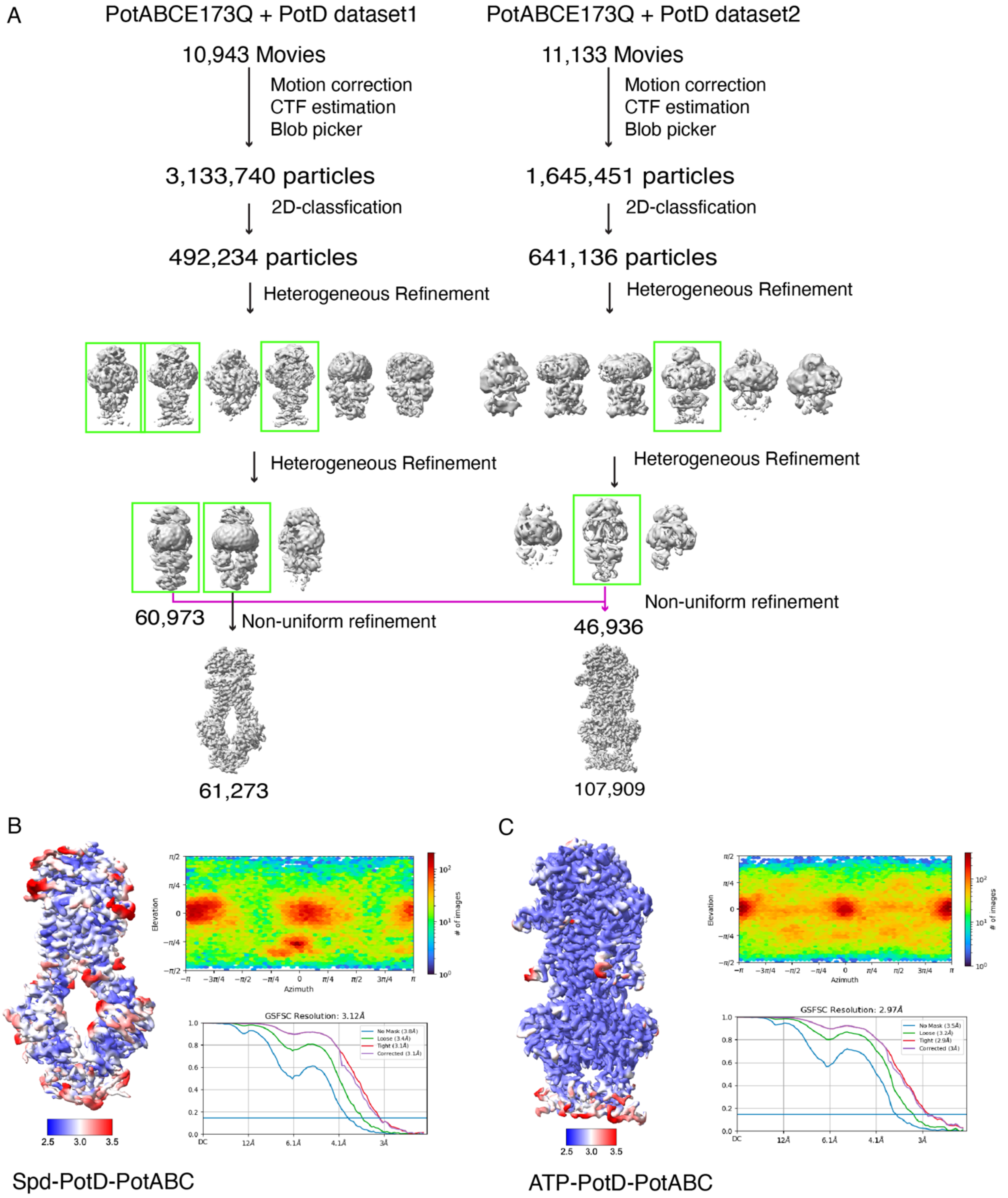

Fig. S6. Cryo-EM data processing of the PotD-PotABC E173Q datasets.

A. The workflow of the PotD-PotABC E173Q datasets 1 and 2 processing. B. Local resolution estimation of the Spd-bound PotD-PotABC electron density map. The angular distribution and the final FSC curves are shown on the right. C. Local resolution estimation of the ATP-bound PotD-

PotABC electron density map. The angular distribution and the final FSC curves are shown on the right.

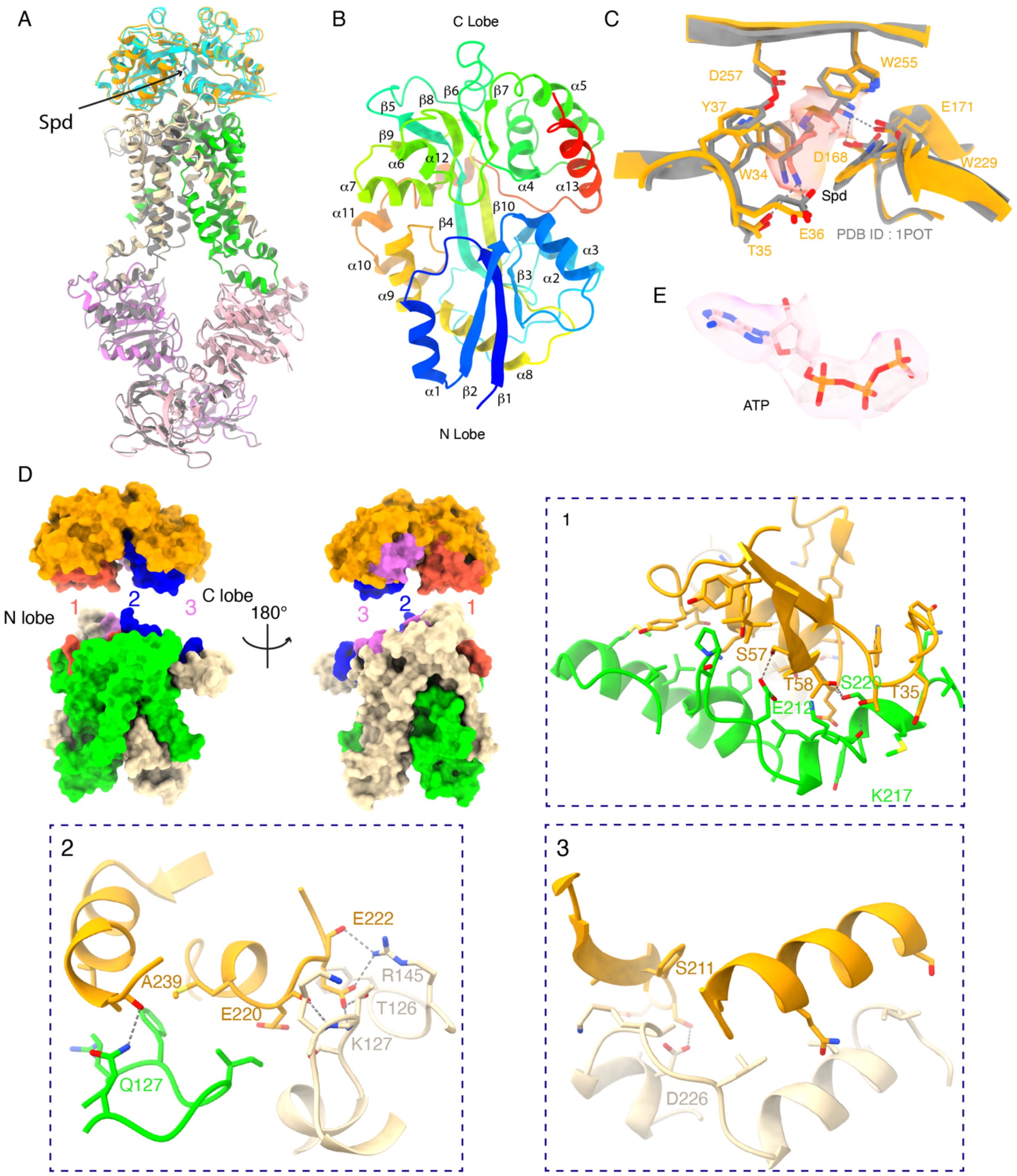

Fig. S7. Structure of the PotD-PotABC complex.

A. The crystal structure of the Spd-bound PotD (PDB ID: 1POT) colored in cyan is superimposed to the Spd-bound PotD-PotABC complex structure. The apo form PotABC structure colored gray is also superimposed to the same Spd-bound PotD-PotABC structure. The bound Spd molecule is indicated by a black arrow. B. Structure of the Spd-bound PotD in the PotD-PotABC complex colored gradually from the N (blue) to the C terminus (red). The secondary structure elements of

PotD are labeled. C. The detailed interaction between Spd and PotD. The crystal structure of the isolated Spd-bound PotD (PDB ID: 1POT) colored in gray is superimposed to that of the Spd bound PotD in the PotD-PotABC complex. The hydrogen bonds are shown as dashed lines. The interacting PotD residues and Spd are shown as sticks. The electron density map of Spd is shown as salmon surface. D. The three interfaces show the interactions between PotD and PotBC in the translocation intermediate state of the PotD-PotABC complex. This panel is presented in a similar way to that in Fig. 2B-2E. E. The electron density map corresponding to the ATP molecule in the ATP-bound structure of PotD-PotABC.

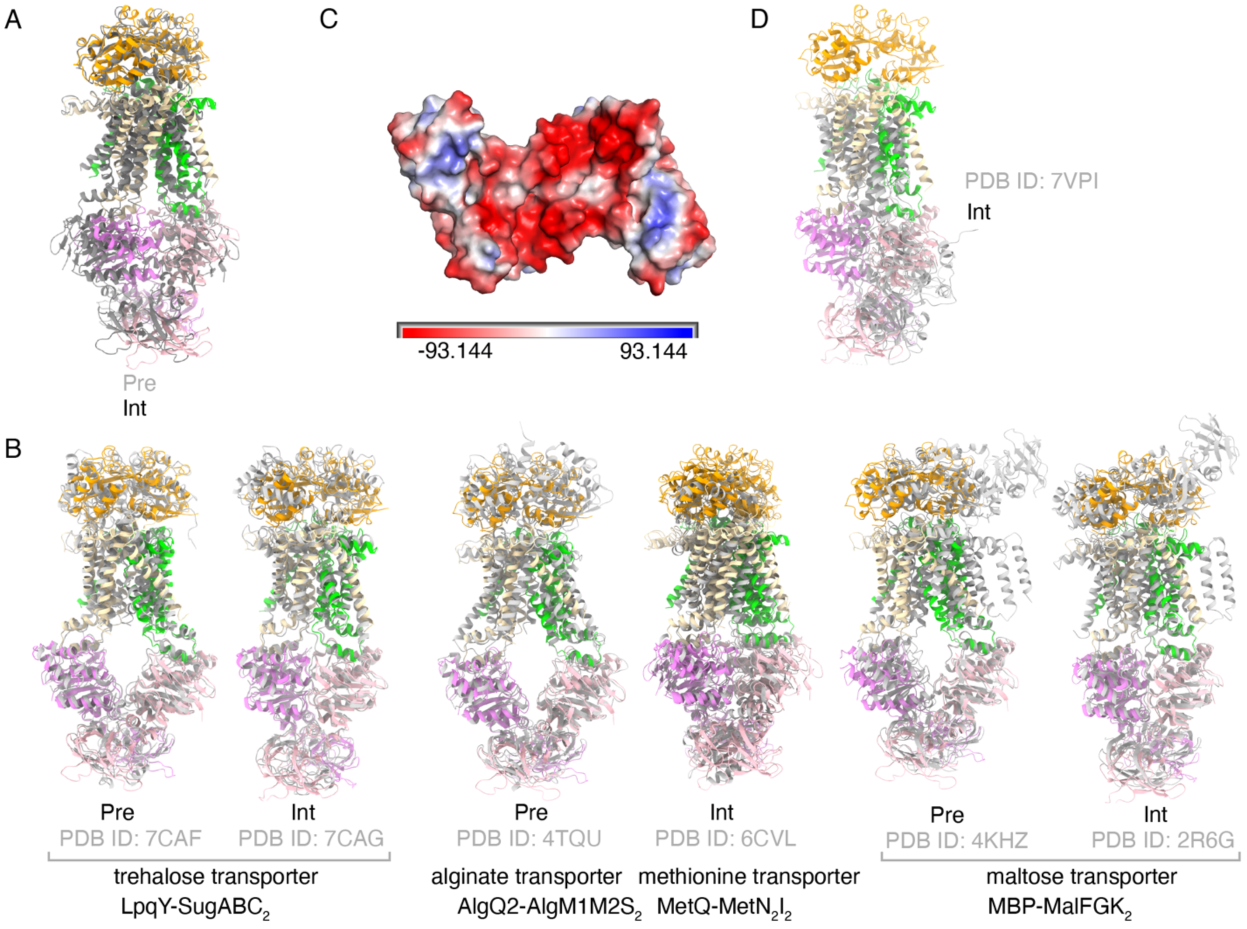

Fig. S8. Structural comparisons and electrostatic potential of the regulation domain of PotA. A. The structural comparison between the PotD-PotABC pre-translocation state (Pre) colored gray and translocation intermediate state (Int). B. The structures of trehalose transporter LpqY-$SugABC_2$ (PDB ID: 7CAF inward-facing, ; 7CAG outward-facing), alginate transporter AlgQ2-$AlgM1M2S_2$ (PDB ID: 4TQU inward-facing), methionine transporter MetQ-$MetN_2I_2$ (PDB ID: 6CVL outward-facing), and maltose transporter MBP-$MalFGK_2$ (PDB ID: 2R6G outward-facing; 4KHZ inward-facing) are colored gray and aligned with PotD-PotABC structures in pre-translocation state or translocation intermediate state. C. The electrostatic potential of the regulation domain of PotA. The extensive negatively charged area (highlighted in red) likely involved in Spd binding. Blue color indicates positively charged surface regions. D. Structural comparison of the PotD-PotABC complex in the translocation intermediate state and the human polyamine transporter ATP13A2 (PDB ID: 7VPI) colored gray.

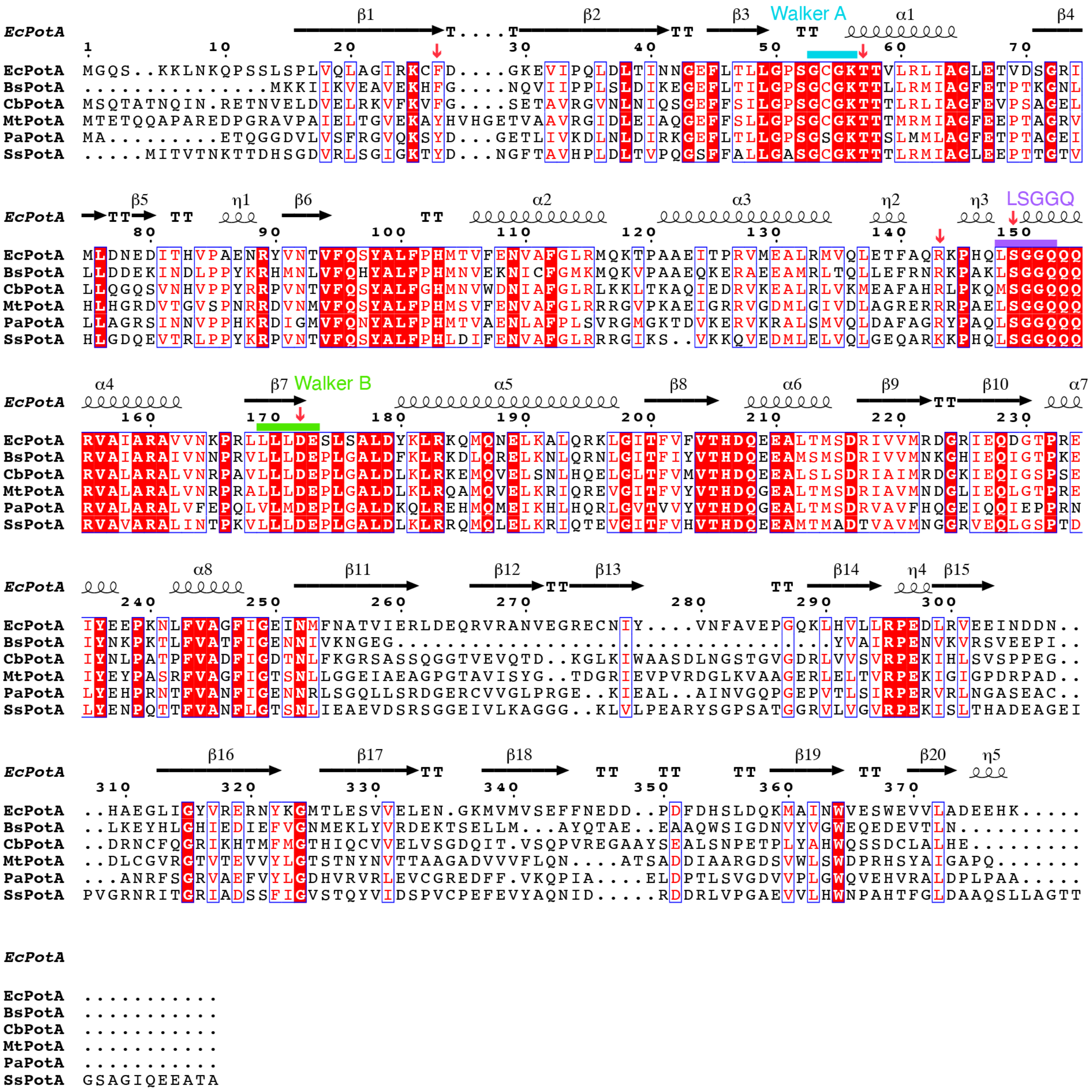

Fig. S9. Multiple sequence alignment of PotA proteins.

The PotA sequences from *Escherichia coli*, *Bacillus subtilis*, *Mycobacterium tuberculosis*, *Streptomyces scabiei*, Cyanobacteria bacterium UBA11691, and *Pseudomonas aeruginosa*, are subjected to multiple sequence alignment by Clustal Omega and Espript 3.0. The functionally important Walker A motif, LSGGQ motif, and Walker B motif are marked with cyan, purple, and green bars, respectively. The residues selected for mutagenesis study are indicted by red arrows.

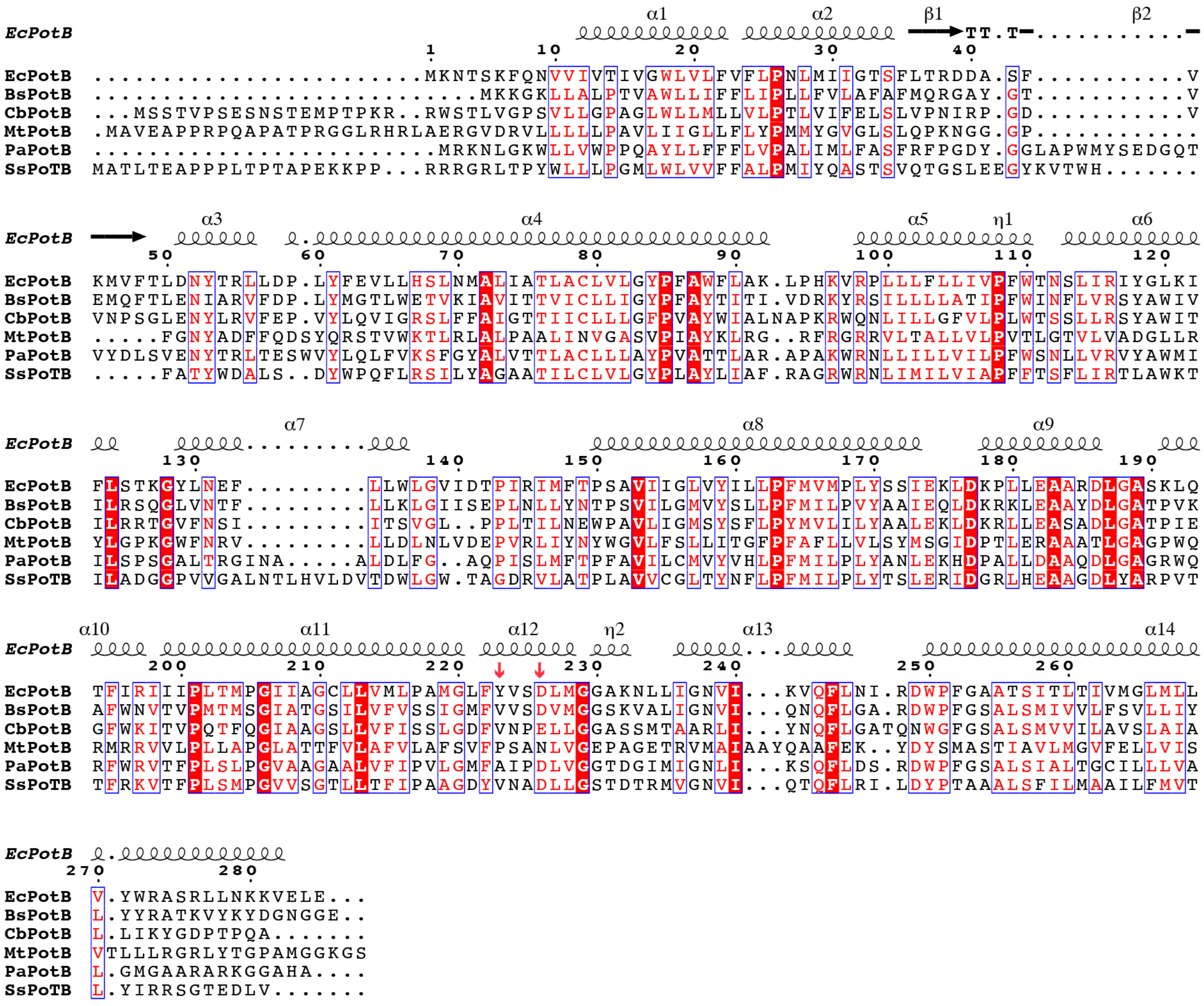

Fig. S10. Multiple sequence alignment of PotB proteins.

The PotB sequences from *Escherichia coli, Bacillus subtilis, Mycobacterium tuberculosis, Streptomyces scabiei,* Cyanobacteria bacterium UBA11691, and *Pseudomonas aeruginosa*, were subjected to multiple sequence alignment by Clustal Omega and Esprit 3.0. The residues selected for mutagenesis study are labeled with red arrows.

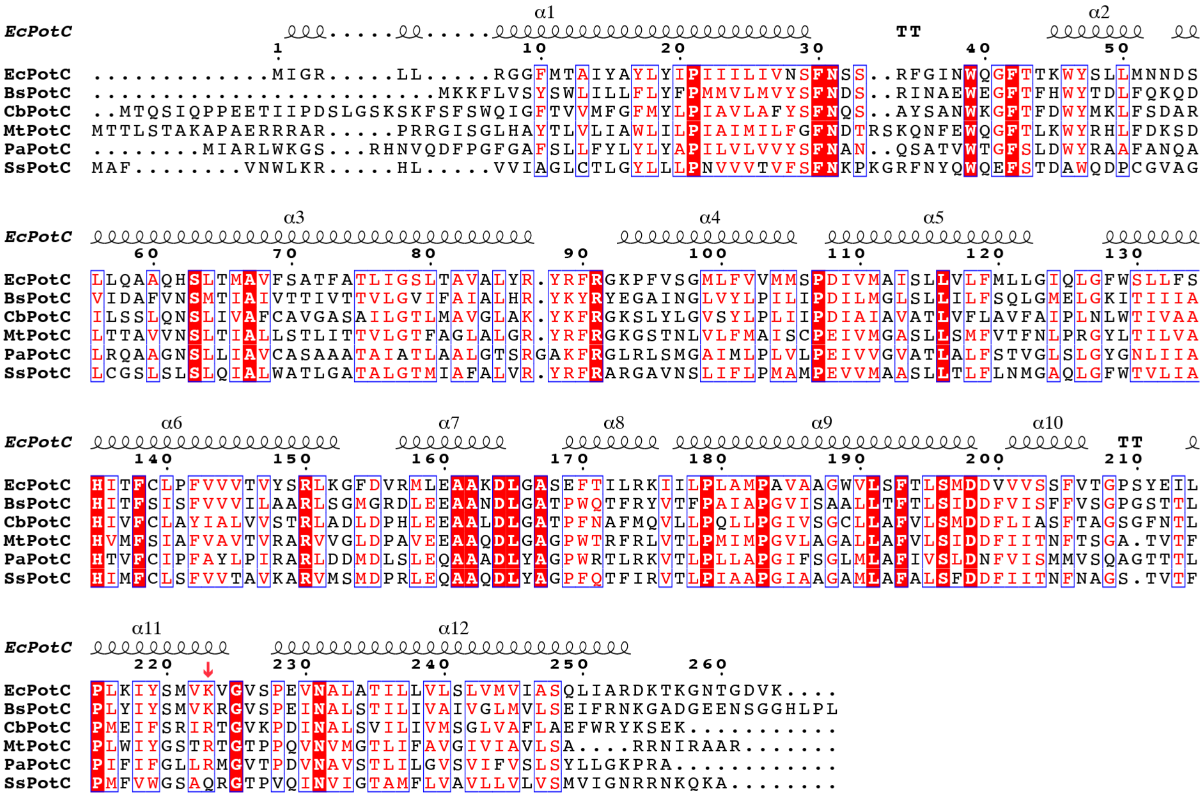

Fig. S11. Multiple sequence alignment of PotC proteins.

The PotC sequences from *Escherichia coli, Bacillus subtilis, Mycobacterium tuberculosis, Streptomyces scabie*i, Cyanobacteria bacterium UBA11691, and *Pseudomonas aeruginosa*, were subjected to multiple sequence alignment by Clustal Omega and Espript 3.0. The residues selected for mutagenesis study are labeled with red arrows.

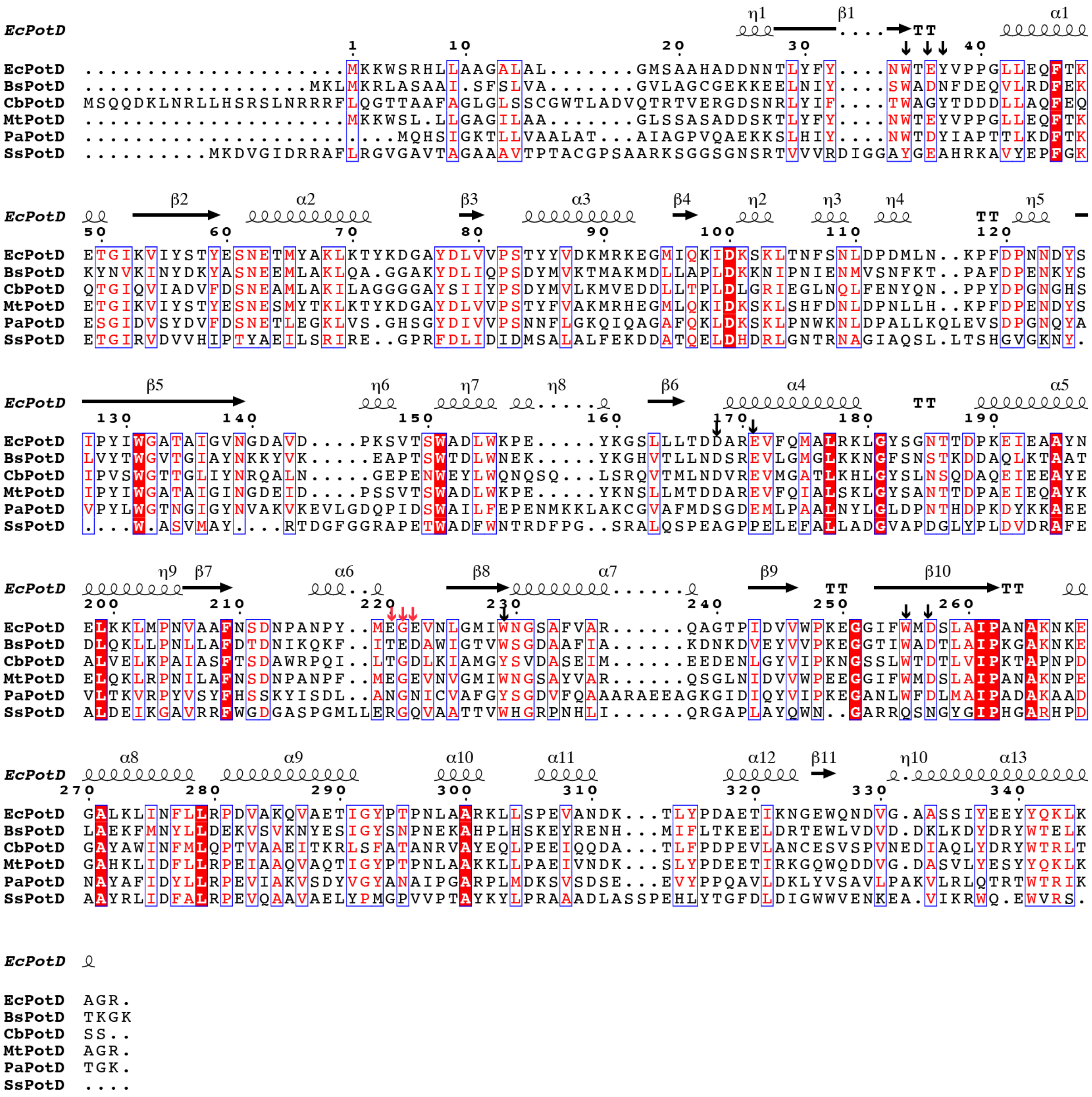

Fig. S12. Multiple sequence alignment of PotD proteins.

The PotD sequences *from Escherichia coli, Bacillus subtilis, Mycobacterium tuberculosis, Streptomyces scabiei*, Cyanobacteria bacterium UBA11691,and *Pseudomonas aeruginosa*, were subjected to multiple sequence alignment by Clustal Omega and Espript 3.0. The residues important for Spd binding are labeled with red arrows. The residues selected for mutagenesis study are labeled with red arrows.

Table S1. Cryo-EM data collection, processing, and model refinement of the structures reported in this study.

	PotABC	Spd-PotABC	Spd-PotD-PotABC	ATP-PotD-PotABC	PotABC nanodisc
PDB ID	8Y5F	8Y5G	8Y5H	8Y5I	8ZX1
EMDB ID	EMD-38933	EMD-38934	EMD-38935	EMD-38936	EMD-60536
Magnification	165,000	165,000	165,000	165,000	130,000
Voltage (kV)	300	300	300	300	300
Electron exposure (e–/Å2)	50	50	50	50	40
Defocus range (μm)	-0.5 ~ -1	-0.5 ~ -1	-0.5 ~ -1	-0.5 ~ -1	~0.6 ~ -1
Pixel size (Å)	0.76	0.76	0.76	0.76	0.97
Symmetry imposed	C1	C1	C1	C1	C1
Initial particle images (no.)	2,300,504	2,300,504	3,133,740	1,645,451	3,767,473
Final particle images (no.)	90,050	98,494	61,273	107,909	68,331
Map resolution (Å)	3.13	3.06	3.12	2.97	3.51
FSC threshold	0.143	0.143	0.143	0.143	0.143
Refinement					
Initial model used (PDB ID)	1POT				
AlphaFold database ID	AF-P69874-F1, AF-P0AFK4-F1, and AF-P0AFK6-F1				
Model composition					
Non-hydrogen atoms	9843	9871	12402	12374	9846
Protein residues	1240	1240	1562	1553	1240
Ligands	0	6	1	4	0
Validation					
B factors (Å2)					
Protein	58	57.17	64.79	54.01	192.24
Ligand		50.57	51.15	54.84	
R.m.s. deviations					
Bond lengths (Å)	0.003	0.006	0.003	0.004	0.007
Bond angles (°)	0.624	0.671	0.657	0.684	1.081
MolProbity score	1.7	1.76	1.71	1.81	2.02
Clashscore	5.26	4.09	7.07	7.5	17.04
Poor rotamers (%)	1.48	1.94	1.03	0.52	0
Ramachandran plot					
Favored (%)	95.77	95.04	95.55	94.04	95.85
Allowed (%)	4.15	4.96	4.45	5.96	4.15
Disallowed (%)	0	0	0	0	0
